# Abstracts from the 25th Italian Congress of Cystic Fibrosis and the 15th National Congress of Cystic Fibrosis Italian Society: Assago, Milan. 10 - 12 October 2019

**DOI:** 10.1186/s13052-020-0790-z

**Published:** 2020-04-01

## S1 Tools to support quality of life in chronic diseases: perspectives in Cystic Fibrosis

### Cristina Rapisarda Sassoon (cristina.rapisarda@gmail.com)

#### Yoga Acharya, Professor of Yoga, Global Trends srl, Milan, Italy

Regular physical activity has been recognized for many years as a key health factor by the scientific community. The focus is particularly high in the evaluation of quality of life perspectives of individuals with chronic disease (HRQoL), therefore also in FC. Recent studies have included a number of disciplines that combine physical and mental training.

Among these, Yoga is particularly interesting since it integrates physical exercise and stress management. To date, the main area of study are: mental health (anxiety, depression, PTSD), cardiovascular diseases, respiratory diseases (asthma and COPD), musculoskeletal disorders and oncological diseases. Among COPD patients, Yoga has proven to be effective in improving exercise capacity, respiratory function and QoL. Among children with chronic asthma, Yoga was found to be effective to reduce drug dependency, lower persistence of asthma symptoms and promote better asthma control. Two studies are currently available in FC, which have formulated the following conclusions: a personalized and individual Yoga program can be considered safe, well tolerated and effective in relation to the respiratory domain of CFQ-R, improvement of joint pain and reduction of anxiety in patients with mild to moderate CF (children and adolescent) [1,2]. The data collected to now, although quantitatively insufficient, encourage further experimentation. Yoga, as a complementary therapy for CF patients, can in fact contribute to: strengthening muscles (respiratory and pelvic floor), improving posture (trunk and rib cage), reducing joint pain, relaxing body-breathing-mind. Above all, the effectiveness of Yoga in CF should be evaluated in relation to its specific ability to control the mind, calming it and making it efficient in anxiety and stress management. Yoga practice develops self-esteem, as well as a positive aptitude to self-control. It also educates in self-discipline, thus training in adherence. A regular Yoga practice can help the CF patient to accept and make constructive a state of health and a therapeutic routine that keeps him or her constantly at a safe distance from the quality of life line of healthy people. Yoga can also be effective in supporting caregivers, whose emotional burden and the resulting states of anxiety and depression are today recognized by numerous studies as a cause of lower adherence to therapies and thus worsening the health conditions of CF patients. Yoga experimentations for caregivers are already underway in oncology. The perspectives in CF could be to make patients and caregivers share the same Yoga practice, and/or to create specific distance tools for caregivers, making them accessible online.

**References**

1. Ruddy J et al. Yoga as a Therapy for Adolescents and Young Adults With Cystic Fibrosis: A Pilot Study. Glob Adv Health Med 2015; 4:32-6.

2. McNamara C et al. Yoga Therapy in Children with Cystic Fibrosis Decreases Immediate Anxiety and Joint Pain. Evid Based Complement Alternat Med 2016; 9429504.

## Genetics / epidemiology

### A1 Cystic fibrosis with residual function mutations: epidemiology and factors influencing lung disease

#### Donatello Salvatore^1,2^, Barbara Giordani^2,3^, Rita Padoan^2^, Annalisa Amato^2,3^, Gianluca Ferrari^2,4^, Fabio Majo^2,5^, Fabiola De Gregorio^1^, Carmela Colangelo^1^

##### ^1^CysticFibrosis Center, AOR Ospedale San Carlo, Potenza, Italy; ^2^Italian Cystic Fibrosis Registry, Rome, Italy; ^3^ Lega Italiana Fibrosi Cistica ONLUS, Rome, Italy; ^4^Centro Nazionale Malattie Rare, Istituto Superiore di Sanità, Rome, Italy; ^5^CysticFibrosis Center, Ospedale Pediatrico Bambino Gesù, Rome, Italy

###### **Correspondence:** Donatello Salvatore (saverdon@gmail.com)

**Background**

Patients with CF and Residual Function mutations *(RFm)* have often delayed diagnosis, milder lung disease and pancreatic sufficiency, compared with homozygous for *F508del (FF).* Here we wish to characterize Italian patients with *RFm* and describe the factors influencing the lung disease, comparing with patients *FF.*

**Materials and methods**

Data from the 2017 Italian Cystic Fibrosis Registry (ICFR) were retrieved for patients carrying *RFm* and with *FF* genotype. Furthermore, percent predicted (pp) FEV_1_was analyzed over a 2-year period (2015 to 2017).

**Results**

904 subjects (452 males) with *RFm* were identified over a total of 5,563 (16.3%) with a median age of 25.5 years (IQR 11.4 – 42.4) and a median age at diagnosis of 5.9 (IQR 0.25 – 23.5). The mean BMI z-score was 0.3 (SD 1.1) in children and BMI 23.4 (SD 4.1) kg/m^2^ in adults. Lung function was characterized by a mean ppFEV_1_of 84.2 (SD 23.8). Prevalence of chronic *Pseudomonas Aeruginosa (PA) and Staphylococcus Aureus* was 27.1%, and 49.7%, respectively. The most frequent complication was liver disease (14.4%), whereas diabetes was rare (3.2%). *FF* patients reported significant lower age at diagnosis, worse nutritional status and lung function, and higher prevalence of complications compared to patients with *RFm*.

The multivariate analysis on lung function outcome showed:

1. Among subjects with *RFm*, strong evidence (p<0.001) of association between lung function and adult age. Evidence of worse lung function was also found among patients with chronic *PA* (-16.9, 95% CI: -16.3 to -13.4). Weak evidence of difference in lung function was found between females and males (-3.0, 95% CI: -6.1 to 0.0, *p=0.052*).
2. Among *FF* subjects lung function was also negatively associated to increasing age from adolescence and to the colonization by *PA*. Lung function was not significantly different between females and males.
3. Patients with genotype *3849+10kbC>T/F508del* reported significant worse lung function compared to all *RF* patients (-11.2, 95% CI: -16.9 to -5.4).

The longitudinal analysis showed no change over the period 2015–2017 in the ppFEV_1_% trend among *RF* and *FF* patients.

**Conclusions**

Patients with CF and *RFm* are numerous in Italy and have a milder clinical phenotype than *FF*. Lung disease of *RF* subjects is more evident since the early adult age and strongly associated to the colonization by *PA. RF* but not *FF* females might report more severe lung disease, but this issue needs further investigation. Patients with the genotype *3849+10kbC→T/F508del* showed more severe lung disease.

All patients provided informed written consent for data publication.

### A2 Clinical features of patients carrying the 5T;TG12 variant in combination with a CF-causing mutation or a CFTR variant at Ancona CF centre

#### Natalia Cirilli, Benedetta Fabrizzi, Nicole Caporelli, Federica Masseria, Andrea Benigni, Marco Cipolli

##### Cystic Fibrosis Centre, Mother-Child Department, United Hospitals, Ancona, Italy

###### **Correspondence:** Natalia Cirilli (natalia.cirilli@ospedaliriuniti.marche.it)

**Background**

The 5T;TG12 variant has varying clinical consequences. Due to this variability it is recommended that clinical criteria alone be used to determine whether a person with this variant has CF (www.cftr2.org).

**Cases report**

At Ancona CF Centre since 2006 27 cases carrying the 5T;TG12 variant in combination with a CF-causing mutation or CFTR variant were identified (12 females; 15 males; median age: 9.6 years; median age at diagnosis: 1.9 years). 3 were identified due to male infertility in adult age; 3 were identified due to CF like respiratory symptoms (bronchiectasis); 22 (81.4%) were identified because positive to CF newborn screening. The first diagnostic label for 19/27 (70.3%) was CFSPID: 6/19 (31.5%) became CF due to new CF like respiratory features and/or 2 or more consecutive positive sweat chloride tests (age range at CF diagnosis: 7 months – 126 months). 6/27 (22.2%) were labeled as CF; 1 was labeled as CBAVD, 1 was labeled as CFTR-RD (Table 1). 7 out of 27 (25.9%) were lost to follow up.

**Conclusion**

The cohort of patients with 5T;TG12 variant in combination with a CF-causing mutation is heterogeneous. The sweat chloride can remain in the borderline range for a long period of time. In our CF centre 31.5% of CFSPID became CF: the time frame to CF diagnosis should be longer than 10 years. Based on our experience we confirm that the clinical picture should lead a personalized strategy to treat this cohort of patients.

Informed consent was obtained from all patients for data publication.

**Table 1 (abstract A2).** Genotypes, sweat chloride (SC) values and clinical features of this cohort of patients

Gender	1^**st**^ mutation	First SC	Last SC	SC min	SC max	Last FEV1	Clinical features
M	N1303K	110	62	61	110	ND	pancreas sufficient; normal nutritional status; intermittent MSSA
F	2789+5G->A	46	49	42	49	89	pancreas sufficient; at risk of obesity; intermittent MSSA
M	[delta]F508	30	52	30	54	90	pancreas sufficient; at risk of obesity; intermittent MSSA
F	2789+5G->A	85	49	40	85	ND	pancreas sufficient; at risk of malnutrition
M	2789+5G->A	43	43	36	81	97	pancreas sufficient; malnutrition; chronic MSSA
M	[delta]F508	68	47	47	68	98	pancreas sufficient; overweight; male infertility; intermittent MSSA
M	R334L	72	71	68	72	53	pancreas sufficient; normal nutritional status; 3 episodes of pseudo-Bartter’s syndrome; intermittent PA
M	[delta]F508	24	49	24	49	ND	pancreas sufficient; normal nutritional status
M	[delta]F508	32	116	32	116	ND	pancreas sufficient; normal nutritional status
M	N1303K	29	84	29	87	ND	pancreas sufficient; malnutrition; intermittent MSSA
M	[delta]F508	34	93	31	93	ND	pancreas sufficient; at risk of malnutrition; intermittent MSSA
F	621+1G->T	25	45	25	64	ND	pancreas sufficient; at risk of malnutrition; intermittent MSSA
M	621+1G->T	39	50	39	50	ND	pancreas sufficient; normal nutritional status
M	621+1G->T	24	57	24	57	ND	pancreas sufficient; at risk of obesity; recurrent bronchitis
M	L997F	16	36	16	36	93	pancreas sufficient; normal nutritional status; bronchiectasis
F	[delta]F508	60	34	32	63	98	pancreas sufficient; normal nutritional status; recurrent respiratory infections
F	[delta]F508	24	55	20	55	102	pancreas sufficient; at risk of obesity; intermittent MSSA
F	G542X	47	57	47	57	62	pancreas sufficient; normal nutritional status; bronchiectasis; kidney stones; hypertension
F	N1303K	36	80	36	80	ND	pancreas sufficient; normal nutritional status
F	[delta]F508	37	35	26	52	ND	pancreas sufficient; normal nutritional status
F	[delta]F508	22	45	22	54	83	atelectasis; normal nutritional status; bronchiectasis
M	W1282X	21	48	21	136	95	pancreas sufficient; normal nutritional status
M	[delta]F508	99	94	94	102	82	pancreas sufficient; obesity; type 2 diabetes mellitus; bladder cancer (surgery in 2011); male infertility
F	[delta]F508	26	60	26	68	ND	pancreas sufficient; normal nutritional status
M	G542X	76	64	64	76	108	pancreas sufficient; overweight; CBAVD
F	[delta]F508	26	157	26	157	101	pancreas sufficient; normal nutritional status; recurrent respiratory infections
F	G85E	34	39	34	39	ND	pancreas sufficient; normal nutritional status

M=male; F=female; ND=not determined; MSSA=methicillin sensible Staphylococcus aureus; PA=Pseudomonas aeruginosa; CBAVD=congenital bilateral aplasia of the vas deferens

### A3 Disease progression and burden in patients with cystic fibrosis homozygous for *F508del* across Europe in an observational registry (VOICE study)

#### Carlo Castellani^1^, Edward F McKone^2^, Harry Heijerman^3^, Amparo Sole^4^, Siobhán B Carr^5^, Jane C Davies^5,6^, Rainald Fischer^7^, Celeste Barreto^8^, Isabelle Fajac^9^, Zheng (Jason) Yuan^10^, Nils Kinnman^10^

##### ^1^Cystic Fibrosis Centre, IRCCS Istituto Giannina Gaslini, Genoa, Italy; ^2^St Vincent’s University Hospital and University College Dublin, Dublin, Ireland; ^3^Department of Pulmonology, University Medical Center Utrecht, Utrecht, the Netherlands; ^4^Adult Cystic Fibrosis and Lung Transplant Unit, University and Polytechnic Hospital La Fe, Universitat de València, Valencia, Spain; ^5^Department of Respiratory Paediatrics, Royal Brompton & Harefield NHS Foundation Trust, London, United Kingdom; ^6^National Heart and Lung Institute, Imperial College London, London, United Kingdom ^7^Lungenheilkunde München-Pasing, Munich, Germany; ^8^Centro Hospitalar Universitário Lisboa Norte, Lisbon, Portugal; ^9^Assistance Publique-Hôpitaux de Paris, Adult Cystic Fibrosis Center, Cochin Hospital, Paris, France and Sorbonne Paris Cité, Université Paris Descartes, Paris, France; ^10^Vertex Pharmaceuticals Incorporated, Boston, MA, USA

###### **Correspondence:** Carlo Castellani (carlocastellani@gaslini.org)

**Background**

The study objective was to assess the disease progression and burden of cystic fibrosis (CF) in patients aged≥ 6 years homozygous for *F508del* in Europe.

**Materials and methods**

VOICE was an observational, encounter-based registry study of patients aged ≥6 years homozygous for *F508del*conducted at 37 CF centres in 7 countries in Europe. Data from 24 months before enrolment were collected retrospectively. Available data from the most recent 24-month period with no CFTR modulator therapy were analysed for all enrolled patients who had not participated in other Vertex studies previously or concurrently. The rate of change in percent predicted forced expiratory volume in 1 second (ppFEV_1_) was estimated by mixed-effect model for repeated measures analysis. Pulmonary exacerbations (PEx; clinician defined), hospitalisations and intravenous (IV) antibiotic treatment were summarised descriptively.

**Results**

889 patients from Italy, the Netherlands, Spain, the UK, Ireland, Germany and Portugal were included (mean age [standard deviation], 22.6 years [11.8]). The observed annualised rate of change in ppFEV_1_ (95% confidence interval, CI) was −2.17 (−2.77 to −1.58) percentage points for all patients aged ≥6 years at the start of the analysis period (n=822), with the steepest rate of decline in patients aged 12–17 years (n=158; −3.92 [−5.03 to −2.81]). The observed rate of change in ppFEV_1_ (95% CI) was −2.06 (−3.23 to −0.90) percentage points in patients aged 6–11 years (n=196) and −1.46 (−2.27 to −0.64) percentage points in patients aged ≥18 years (n=468). The observed annualised PEx rate was 0.85 for PEx overall, 0.37 for PEx requiring hospitalisations and 0.42 for PEx requiring IV antibiotics (Table 1). PEx and hospitalisations were more common in patients with worse baseline lung function.

**Conclusions**

Findings reinforce the significant disease progression and burden in a European population homozygous for *F508del*.

**Sponsor:** Vertex Pharmaceuticals Incorporated.

**Table 1 (abstract A3).** Summary of PEx and hospitalisations by baseline ppFEV_1_subgroups

Observed event rate per patient-year, n (%)	Overall (N=889)	Baseline ppFEV_**1**_<40% (n=114)	Baseline ppFEV_**1**_ ≥40%–<70% (n=281)	Baseline ppFEV_**1**_ ≥70%–≤90% (n=248)	Baseline ppFEV_**1**_>90% (n=227)
**All PEx**	491 (55.2) **0.85**	84 (73.7) **1.39**	180 (64.1) **1.08**	126 (50.8) **0.66**	94 (41.4) **0.58**
**PEx requiring hospitalisations**	296 (33.3) **0.37**	64 (56.1) **0.83**	117 (41.6) **0.52**	67 (27.0) **0.24**	42 (18.5) **0.15**
**PEx requiring IV antibiotics**	327 (36.8) **0.42**	69 (60.5) **0.97**	133 (47.3) **0.60**	73 (29.4) **0.28**	46 (20.3) **0.17**
**Hospitalisations**	386 (43.4) **0.57**	79 (69.3) **1.30**	142 (50.5) **0.78**	90 (36.3) **0.38**	69 (30.4) **0.28**

## New therapies and outcome measures

### A4 Synthesis and biological characterization of new metalloproteinase inhibitors for reducing lung inflammation in cystic fibrosis patients

#### Grazia Lombardi^1^, Silvia Fallarini^1^, Enza Torre^1^, Marco Fragai^2,3^, Marco Martinucci^4^, Cristina Nativi^2^

##### ^1^Depatment of Pharmaceutical Sciences, University of “Piemonte Orientale Amedeo Avogadro”, Novara, Italy; ^2^Department of Chemistry “Ugo Schiff”, University of Florence, Florence, Italy; ^3^CeRM, University of Florence, Florence, Italy; ^4^Giotto Biotech, Florence, Italy

###### **Correspondence:** Grazia Lombardi (grazia.lombardi@uniupo.it)

**Background**

Matrix metalloproteases (MMPs) are proteolytic enzymes restoring tissue architecture following injury. The importance of MMP activity in cystic fibrosis (CF) pathogenesis has been demonstrated. Particularly, the MMP-9 activity increases in CF patients undergoing acute exacerbation, and this correlates with the degradation of its natural tissue inhibitor. The reduction of MMP-9 activity in CF patients by MMP inhibitors (MMPIs) represents an attracting target, although this effort has been largely unsuccessful, mainly due to lack of appropriate drug delivery in the lungs, adverse effects, and poor enzymatic selectivity of compounds. The aim is to synthesize, purify, and fully characterize a new family of MMPIs, armed to be linked to carrier proteins and to determine their biological properties

**Materials and methods**

The synthesis of new MMPIs relies on a gram-scale synthetic strategy previously optimized [1,2], starting from commercially available glycine and inserting on the aminoacidic nitrogen the pre-formed spacer via Mitzunobu reaction. The terminal carboxylic group of the spacers will be activated as N-hydroxysuccinimide for protein scaffold conjugation. All intermediates will be purified by chromatography/dialysis and fully characterized by NMR/MS/MALDI-MS. The biocompatibility and immunogenicity of each compound will be tested on human peripheral blood mononuclear cells (PBMCs)by Trypan blue/MTT assays and cell proliferation/IL-2 measurements [3]. The ability of each compound to inhibit MMP-9 activity will be measured on *ex vivo* human neutrophils by succinylated-gelatin assay.

**Results**

We developed and characterized a new family of MMPIs soluble in water.No compounds induced cytotoxicity at 12-24 h, demonstrating their fully biocompatibility. Cell exposure to each compound did not stimulate immune cell activation, ruling out possible immunogenicity. The ability of the compounds to inhibit of MMP-9 activity was at low nanomolar range, suggesting a good enzymatic specificity.

**Conclusions**

New biocompatible, no immunogenic, MMP-9 inhibitors, soluble in water and structurally useful to decorate endogenous proteins, were here described.

**References**

1. Bertini I, Calderone V, Fragai M et al. Exploring the subtleties of drug-receptor interactions: the case of matrix metalloproteinases. J Am Chem Soc. 2007;129:2466-75

2. Bartoloni M, Domínguez BE, Dragoni E et al. Targeting matrix metalloproteinases: design of a bifunctional inhibitor for presentation by tumour-associated galectins. Chemistry. 2013;19:1896-902

3. Fallarini S, Buzzi B, Giovarruscio S et al. A Synthetic Disaccharide Analogue from Neisseria meningitidis A Capsular Polysaccharide Stimulates Immune Cell Responses and Induces Immunoglobulin G (IgG) Production in Mice When Protein-Conjugated. ACS Infect Dis. 2015;1:487-96

### A5 Effectiveness of Ivacaftor in severe cystic fibrosis patients and CFTR residual function mutations

#### Donatello Salvatore^1^, Cesare Braggion^2^, Barbara Messore^3^, Giovanna Pisi^4^, Maria Adelaide Calderazzo^5^, Giovanna Pizzamiglio^6^, Fabio Majo^7^, Federico Cresta^8^, Antonio Manca^9^, Michela Francalanci^2^, Vito Terlizzi^2^, Carmela Colangelo^1^, Fabiola De Gregorio^1^, Francesco Longo^4^, Elisa Clivati^3^, Carlotta Biglia^3^, Mimma Caloiero^5^, Matteo Botti^10^

##### ^1^Cystic Fibrosis Center, AOR Ospedale San Carlo, Potenza, Italy; ^2^Cystic Fibrosis Center, Anna Meyer Children's University Hospital, Florence, Italy; ^3^Adult CysticFibrosis Center, Azienda Ospedaliera Universitaria San Luigi Gonzaga, Orbassano, Italy; ^4^Cystic Fibrosis Unit, Department of Pediatrics, University Hospital, Parma, Italy; ^5^CysticFibrosis Center, Hospital Giovanni Paolo II, Lamezia Terme, Italy; ^6^CysticFibrosis Center - Adult Unit, Fondazione IRCCS Ca’ Granda, Ospedale Maggiore Policlinico, Milan, Italy; ^7^Cystic Fibrosis Unit, Bambino Gesù Children’s Hospital, Rome, Italy; ^8^Cystic Fibrosis Center, IRCCS G. Gaslini Institute, Genoa, Italy; ^9^Cystic Fibrosis Center, AOU Policlinico Consorziale, Bari, Italy; ^10^Department of Pediatric, Santa Chiara Hospital, University of Pisa, Pisa, Italy

###### **Correspondence:** Donatello Salvatore (saverdon@gmail.com)

**Background**

Ivacaftor is a CFTR potentiator approved in EU for class-III and for residual function (RF) CFTR mutations. Limited data are available on the effects of Ivacaftor on these patients with severe lung disease, usually excluded from RCT. Since 2016, subjects with CF and severe lung disease, with RF mutations, had access to Ivacaftor as compassionate use. We retrospectively describe the effectiveness and safety of this treatment.

**Materials and methods**

25 subjects (2 men). Median age: 46.7 yrs, range 21.3-58.4 yrs; Severe lung disease: Mean (SD) ppFEV1 31.5% (14.5).

- Genotypes:
  - 3849+10KbC-T/ F508del (n=5), 3849+10KbC-T4382delA (n=1);
  - 2789+5G->A /F508del (n=3), 2789+5G->A /M1V (n=2), 2789+5G->A /W1282X (N=2), 2789+5G->A /1602delCT (n=1)
  - D579G /F508del (n=3)
  - D1152H /F508del (n=2), D1152H /D110H (n=1), D1152H /N1303K (n=1)
  - R352Q /Delexon22-23 (n=1), R352Q /F508del (n=1)
  - 3272-26A→G /E585X (n=1); R117C /N1303K (n=1)

Lung function, BMI, use of antibiotics, sweat Cl, and microbiology were evaluated in the 12 months before starting Ivacaftor and every 3 months during the follow up. Mean treatment duration was 11.3 ± 6.9 months. 10/25 pts had therapy for greater than one year.

**Results**

The mean absolute change (MAC) (95% confidence interval [CI]) in ppFEV_1_ from the baseline value was 7.1 (2.3 to 11.9) after 4 weeks (w), 7.8 (2.8 to 12.8) after 12 w, 9.74 (4.1 to 15.4) at 24 w, 8.5 (3.5 to 13.5) after 36 w and 8.6 (5.1 to 12.1) after 48 w. The MAC (95% IC) of BMI was 0.91 kg/m^2^ (-0.17 to 1.99) after 6 months and 1.03 (-0.59 to 2.65) after 12 months. Days of antibiotic therapy decreased by 80% in the first 12 months of follow up as compared to the 12 months before starting Ivacaftor. The MAC (95% IC) of sweat Cl was -19.7 mmol/L (-37.0 to -1.7). Sputum microbiology was unchanged. No safety concerns were registered. Results were independent of the presence of F508del in the genotype.

**Conclusions**

These cases expand our knowledge about potential benefits of Ivacaftor for patients with CF carrying RF mutations with severe lung disease. At this moment, subjects with CF and RF mutations without F508del on the 2^nd^ allele do not have access to therapies with CFTR modulators. These results support the request of SIFC to AIFA of use of Ivacaftor in severely affected subjects with CF and RF mutations without F508del.

All patients provided informed written consent for data publication.

### A6 Phenotype effects by early treatment with Ivacaftor

#### Giuseppina Leonetti^1^, Danila Rosa Iusco^1^, Domenica De Venuto^1^, Patrizia Liberti^1^, Letizia Musolino^2^, Angela Maria Polizzi^2^, Anna Diana^2^, Antonio Manca^1^

##### ^1^Centro Regionale Pugliese Specializzato di riferimento per la Fibrosi Cistica, A.O.U. Consorziale Policlinico di Bari, Bari, Italy^; 2^U.O.C. Laboratorio di Genetica Medica-Settore Fibrosi Cistica, A.O.U. Consorziale Policlinico di Bari, Bari, Italy

###### **Correspondence:** Giuseppina Leonetti (susy.leonetti@virgilio.it)

**Background**

Progress in the survival and quality of life of Cystic Fibrosis patients, until the recent advent of CFTR potentiators and/or modulators depended on the intensity of the diagnostic therapeutic program and the compliance.

**Case report**

The course of a patient with mild respiratory symptoms has been evaluated. In particular, the following parameters were considered: auxological trend, respiratory function tests (FEV_1_, CVF, MMEF), CFQR, respiratory sputum bacteriology. Patient, female, born with natural childbirth at the fortieth week of gestation. APGAR score 9-10, body weight 3110 gr, body height 51 cm. From the age of 7 months , several bronchitic episodes occurred (every 15 days) with mucus resistant to common antibiotic therapies. For suspected GER she followed ranitidine suspension therapy without success. Growth arrests at the age of 15 months. At the age of 18 months the patient performs two sweat test with a positive result. The genetic investigation showed the F508del/G1349D mutations; the fecal elastase was 449 ug/g. She started aerosol therapy with Salbutamol, a rehydration therapy and vitamins. At age 6 years she started treatment with Ivacaftor. After 3 years of follow-up, the patient achieved a notable height and weight increase with BMI from 13 to 17.85. The spirometric indices increased by an average of 40% (Table1). In addition, the patient reported a significant improvement in the quality of life with the eradication of Pseudomonas aeruginosa without exacerbations.

**Conclusion**

The early use of potentiators and/or modulators of CFTR in the presymptomatic period could lead to a better clinical evolution of these patients.

Patient’s parents gave consent for the publication of clinical data.

**Table 1 (abstract A6).** Anthropometric measures, spirometric tests, CFQR, bacteriology over the follow-up

Date	Age	Weight Kg	Height cm	BMI	FEV_1_ %	FVC %	MMEF %	CFQR	Microbiology
12/01/16	6.02	18.1	118	13	56.9	55.9	39.5	64	SA + PA
26/05/16	6.39	21.7	120	15.7	111.4	106.3	98.2	90	SA + PA
15/06/17	7.44	26.8	127.9	16.38	94.9	100.8	71	94	SA
10/07/18	8.51	32	136	17.3	95	89	86	95	SA
12/02/19	9.1	36	142	17.85	101	93	132	91	Neg

SA=Staphylococcus aureus; PA= Pseudomonas aeruginosa

### A7 Cystic fibrosis and new therapeutic strategies: Ivacaftor, a “bridge therapy” towards lung transplantation

#### Francesca Ficili^1^, Valeria Pavone^2^, Annalisa Ferlisi^1^, Gabriella Traverso^1^, Lisa Termini^1^, Maria Antonietta Orlando^1^, Elisa Parisi^2^, Caterina Di Girgenti^3^, Marcella Bertolino^1^, Stafania Barrale^1^, Mirella Collura^1^

##### ^1^Cystic Fibrosis Center and Chronic Respiratory Diseases, O.U. Second Pediatrics “Di Cristina Hospital”, ARNAS CIVICO, Palermo, Italy; ^2^Department of Sciences for the Promotion of Maternal and Child Health “G. D’Alessandro”, University of Palermo, Palermo, Italy; ^3^S.D.O.U. Molecular genetics. ARNAS Civico, Palermo, Italy

###### **Correspondence:** Francesca Ficili (fficili@hotmail.com)

**Background**

Cystic fibrosis (CF) is the most common genetically determined, life limiting disease. The genetic origin of CF is the mutations in the CF transmembrane conductance regulator (CFTR). CFTR mutations may be classified into six categories. Class III mutations consists in defections within chloride channel gating. Ivacaftor is a potentiator that augment chloride transport and increase airway surface liquid height and cilia beat frequency in airway epithelial cells, expressing a CFTR gating mutation.

Lung transplantation is the only therapeutic possibility for CF patients with serious respiratory deficiency. Several parameters of the respiratory function and the general and metabolic state of the patient are evaluated by the Transplant Center in order to define precise clinical criteria to identify the “transplant window”.

**Case report**

We describe the clinical history of C., female, 45 years old. Genetics: DF508/G178R. Pancreatic failure since June 2013. Normal liver and kidney function. On the waiting list since June 2015for bipulmonary transplantation. She has assumed Ivacaftor from September 2015. Patient has been followed up by clinical examination, instrumental and laboratory tests. Auxological parameters and respiratory function were monitored. She was subjected to a quality of life assessment questionnaire: CFQ-R.From the beginning of the therapy program, after about 3 years of treatment, an improvement of the auxometric parameters was observed, resulting in an increase in BMI from 19.9 to 25. Respiratory function has also improved (FEV1 from 19% to 25%). At the sweat test a decrease in chloride concentration from 127 mmol/l to 52 mmol/l has been recorded. Over the years, the patient's perception of the disease has improved with CFQ-R score increasing from 72 to 90. She performed eye and ECG examinations to assess possible toxicity of the drug. No adverse events were reported. In May 2019, the patient underwent a successful bipulmonary transplant. Today she has good general clinical conditions and continues follow-up.

**Conclusion**

As recent studies have shown, Ivacaftor has been found to be a drug that can improve respiratory function, metabolic assesment and consequently the quality of life for the patients with gating mutations. In the case presented, the treatment can be considered a valid “Bridge therapy” as it has allowed to achieve good conditionsfor the bipulmonary transplantation. Ivacaftor is an example of innovative therapeutic strategy for carriers of a CFTR channel gating mutation. The further development of such approaches offers great promise for future therapeutic strategies in CF.

Patient gave consent for the publication of clinical data.

### A8 Real-life experience in a population of Cystic Fibrosis patients with homozygous F508del mutation treated with Lumacaftor/Ivacaftor (LUM/IVA)

#### Giovanna Pisi, Silvia Dioni, Cinzia Spaggiari, Francesco Longo, Maria Candida Tripodi

##### Cystic Fibrosis Unit, Department of Pediatrics, University Hospital, Parma, Italy

###### **Correspondence:** Giovanna Pisi (gpisi@ao.pr.it)

**Background**

Since 2016, CF patients with homozygous F508del had access to LUM/IVA treatment. We retrospectively describe the effectiveness of this treatment, focusing on psychological outcomes.

**Materials and methods**

33 patients homozygous for F508del (19 males), mean age: 30.5 yrs (SD 9.7), range 11-49 years were studied. The mean values of lung function, BMI, sweat Cl, CFQ-R, Patient Health Questionnaire 9 (PHQ-9) and General Anxiety Disorder-7 (GAD-7) were compared in the 12 months before and after starting LUM/IVA. We used the Wilcoxon rank test and paired T Student for non parametrical and parametrical variables, respectively. We considered as statistically significant a p value< 0.05.

**Results**

After treatment with LUM/IVA we observed a significant increase of the mean values for ppFEV_1_ [before 72, SD 18 after 77, SD 17, p<0.002], ppFVC [before 89, SD 11 after 93, SD 12, p<0.01], BMI kg/m^2^ [before 20.7 SD 2.7 after 21.2 SD 2.5, p<0.02]. After LUM/IVA the mean value of Sweat Cl mmol/L significantly decreased from 119 (SD 21) to 84 (SD 19, p< 0.001). Concerning the CFQ-R scores, we found a significantly increase of the mean values for physical functioning [before 73, SD 21 after 78, SD 24, p<0.02], eating [before 87 SD 20 after 93 SD 15, p<0.05] and body image [before 69 SD 26 after 76 SD 22, p<0.03] domains. No significantly differences were detected in PHQ-9 and GAD-7 scores.

**Conclusions**

These real-life experiences in CF patients with F508del homozygous treated with LUM/IVA confirms the effectiveness of the treatment on lung function, BMI and sweat test Chloride. With regard to the psychological-side effects our results prove that the positive influence of LUM/IVA seems to be limited to the domains related to body image and nutrition. Further evaluations are needed to explore the long term effect of LUM/IVA on the mental health and psychological wellbeing.

Patients gave informed consent to data publication.

### A9 Improvement of lung clearance index in a homozygous F508del patient with mild cystic fibrosis in treatment with Lumacaftor/Ivacaftor

#### Giuseppe Fabio Parisi, Maria Papale, Sara Manti, Enza Mulè, Donatella Aloisio, Novella Rotolo, Salvatore Leonardi

##### CysticFibrosis Center Catania, AOU Policlinico Vittorio Emanuele, University of Catania, Catania, Italy

###### **Correspondence:** Giuseppe Fabio Parisi (giuseppeparisi88@hotmail.it)

**Background**

The lung clearance index (LCI) measured by multiple breath washout (MBW) has been shown to be a sensitive measure to capture lung function abnormalities in cystic fibrosis (CF) patients. LCI can also be used to detect treatment effects in interventional trials. Lumacaftor/Ivacaftor is available for CF patients aged12 years and older who are homozygous for the F508del-CFTRbut the timing when starting treatment in patients with mild CF lung disease is sometimes a matter of debate. Here we present the case of a patient with mild CF who started therapy with Lumacaftor/Ivacaftor using LCI to assess the response to treatment.

**Case Report**

13 year-old boy, genotype F508del/F508del, intermittent methicillin-sensitive *Staphylococcus aureus* colonization, last chest tomography (CT) showed some small central bronchiectasis, the forced expiratory volume in 1 s (FEV1) was 93% of predicted, body mass index (BMI) -1.18 SDS. We performed MBW test which showed a LCI of 9.51. In consideration of the aforementioned clinical features, we decided to enhance inhaled therapy, by starting the mucolytic Dornase Alfa, and to initiate therapy with Lumacaftor/Ivacaftor. At baseline, he had a total distance walked during the Six Minute Walk Test (6MWT) of 420 meters, a Cystic Fibrosis Questionnaire-Revised (CFQR) score for respiratory symptoms of 85, one pulmonary exacerbations during the previous year and a level of chloride of 111 mEq/l in the sweat test. After 12 months of treatment, we observed an improvement in all the clinical features as well as a reduction in LCI which was 7.25 (-23.7% than the baseline), as shown in Table 1.

**Conclusion**

Our case demonstrates how LCI can be useful in discriminating patients with mild CF lung disease to evaluate the start of therapy with modulators. Furthermore, LCI could be considered a useful tool to assess the response to a treatment in CF. A relative change of greater than 15% between visits, as in our case, is likely outside the intrinsic variability of the test and it is considered physiologically relevant.

Patient’s parents gave consent for the publication of clinical data.

**Table 1 (abstract A9).** Outcome measures

	Baseline	After 12 months
FEV1 (% of predicted)	93	96
LCI	9.51	7.25
BMI (SDS)	-1.18	-0.47
6MWT (m)	420	450
CFQR	85	100
Sweat test (chloride mEq/l)	111	106
Pulmonary exacerbations	1	0

### A10 Adherence to Lumacaftor-Ivacaftor in cystic fibrosis patients

#### Luca Cristiani^1^, Fabio Majo^1^, Riccardo Valerio De Biase^1^, Valentina Milano^1^, Federico Alghisi^1^, Enza Montemitro^1^, Daniela Savi^1,2^, Sergio Bella^1^, Vincenzina Lucidi^1^

##### ^1^Cystic Fibrosis Unit, Bambino Gesù Children's Hospital, Rome, Italy; ^2^Adult Cystic Fibrosis Center, Sapienza University of Rome, Italy

###### **Correspondence:** Luca Cristiani (lcristiani1989@gmail.com)

**Background**

Poor adherence rates to treatment is frequently reported in cystic fibrosis (CF). Specific data about CFTR modulators are currently lacking, with only one report showing suboptimal adherence to Ivacaftor [1]. Several methods of adherence assessment have been reported, such as daily phone dairy, self-report, prescription refill history (PRH) and electronic monitoring. In Italy PRH data are hard to collect due to the absence of a network between territorial pharmacies and CF centers. In our region (Lazio, Italy), during 2016-2019 period, Lumacaftor-Ivacaftor (LUM-IVA) was delivered by in-hospital pharmacy, according to specific regional law. This offered us an unique opportunity to assess adherence to LUM-IVA in this window of time using PRH.

**Materials and methods**

We conducted a single-center, retrospective, observational study investigating adherence rates to LUM-IVA in 20 CF patients (11 males, 9 females) homozygous for F508del mutation (median age 20 years, range 10 – 44 years), referred to Bambino Gesù Children’s Hospital (Rome). Observation period spanned from October 2016 to July 2019. PRH data were recorded by in-hospital pharmacy service using Medical Possession Ratio (MPR - total amount of medication obtained by the patient divided by the total amount of prescribed medication). At the end of the observation period, a phone interview was conducted assessing self-reported adherence (0-100%). Spearman coefficient was used to assess correlation between MPR and self-reported adherence.

**Results**

Average monitoring period was 26.7 months (range 5-32 months). Overall, median MPR derived adherence was 97%, ranging from 39% to 107%. Self-reported median adherence was 98.5%, ranging from 50% to 100%. In 4 out of 20 patients (15%) MPR derived adherence was < 65%. No significant correlation was found between MPR and self-reported adherence (r 0.31 p 0.17).

**Conclusions**

To our knowledge, this is the first attempt to assess adherence to LUM-IVA in CF patients. Our results show optimal adherence to LUM-IVA in the majority of our sample. Those results are in contrast with the current literature on adherence and may reflect the specificity of Italian CF population (not yet described elsewhere) and/or patients’ expectations on CFTR modulators. However, a consistent number of patients (4/20) shows a poor – to – suboptimal pattern of adherence. Routine adherence screening should be part of the standard care of CF patients, especially in the era of CFTR modulators. Cost-effectiveness analysis, clinical implications assessment and tailored countermeasures are needed.

Patients or their parents gave consent for the publication of clinical data.

**Reference**

1. Abbott J, Bilton D. Adherence to Ivacaftor is suboptimal. J Cyst Fibros 2015;14:547-8

### A11 Bridging CFTR-modulators to infection by defining the impact of therapies on airway microbiology and clinical response in CF patients

#### Cristina Cigana^1^, Giulia Mancini^1^, Lisa Cariani^2^, Arianna Bisogno^2^, Silvia Bricchi^3^, Claudia Caslini^1^, Daniela Girelli^2^, Beatriz Alcalà-Franco^1^, Medede Melessike^1^, Alessandra Bragonzi^1^, Carla Colombo^2,3^

##### ^1^Infections and Cystic Fibrosis Unit, IRCCS San Raffaele Scientific Institute, Milan, Italy; ^2^Centro Regionale Fibrosi Cistica, Fondazione IRCCS Ca’ Granda, Ospedale MaggiorePoliclinico, Milan, Italy; ^3^Università degli Studi di Milano, Milan, Italy

###### **Correspondence:** Cristina Cigana (cigana.cristina@hsr.it)

**Background**

Cystic Fibrosis Transmembrane conductance regulator (CFTR)-modulators have been approved as a mutation-targeted personalized treatment for CF. Treatment efficacy is variable suggesting that individual factors may further influence drug effectiveness. New approaches that better support the identification of responders to CFTR-modulators are the clinical need. Here, we bridge CFTR-modulators to infection by defining the impact of these therapies on airway microbiology and clinical response in CF patients.

**Materials and methods**

Antimicrobial activity of CFTR-modulators and synergism with standard antibiotics were evaluated on a bio-bank of longitudinal *Pseudomonas aeruginosa* and *Staphylococcus aureus* isolates. Next, a cohort of CF patients homozygous for F508del-CFTR mutation is under investigation in a longitudinal study before and after Lumacaftor/Ivacaftor treatment.

**Results**

None or minimal antimicrobial effect was observed upon exposure of *P. aeruginosa* isolates to CFTR-modulators alone, including high concentrations up to 16 μg/ml for ivacaftor (IVA) and tezacaftor (TEZ) and 256 μg/ml for lumacaftor (LUM). Whereas, synergistic effect of CFTR-modulators with antibiotics was detected. IVA synergized with polymyxin and colistin in almost all *P. aeruginosa* isolates, while it did not show any synergy with other antibiotics. Differently, TEZ and LUM showed few synergies, even at high concentrations. Of note, IVA showed an antimicrobial activity per se against *S. aureus* isolates at clinically relevant concentrations, and synergized with antibiotics. LUM showed a similar activity although at higher concentrations. Conversely, TEZ was completely ineffective. We studied 14 patients, 12 years of age or older, homozygous for the F508del mutation, who had been taking LUMA/IVA for at least 2 years, with particular regard to their airways infections before and after treatment. Pulmonary exacerbations significantly decreased from baseline after both the first and the second year of treatment. Pulmonary function (FEV_1_ % predicted) and body mass index also improved from baseline but not significantly. After the first and second treatment year, no significant change in microbiological isolation was observed. The analysis of clinical data from additional 28 patients is in progress. In addition the antimicrobial activity of CFTR-modulators on patients’ isolates is under investigation.

**Conclusions**

So far, these results suggest that CFTR-modulators can have an anti-bacterial activity and influence antibiotic efficacy through a synergistic effect that varies dependently from the isolate, modifying treatment efficacy. These initial clinical studies support the needs for further evaluation of their impact on infection.

Supported by the Italian Cystic Fibrosis Research Foundation and Fondazione Centro San Raffaele

### A12 A screening system to test novel modulator drugs of *CFTR* gene transcriptional regulation

#### Marika Comegna^1,2^, Federica Tricarico^1,2^, Alessandra Potenza^1,2^, Immacolata Zollo^1,2^, Noemi Falcone^1,2^, Gustavo Cernera^1,2^, Federica Zarrilli^1,2^, Giuseppe Castaldo^1,2^, Felice Amato^1,2^

##### ^1^Department of Molecular Medicine and Biotechnology, University of Naples Federico II, Naples, Italy; ^2^CEINGE- Advanced Biotechnology, Naples, Italy

###### **Correspondence:** Marika Comegna (marika.comegna@unina.it)

**Background**

The Cystic Fibrosis (CF) is an autosomal recessive disease caused by mutations in *CFTR* gene, resulting in alteration of the chloride channel activity. Currently, the therapeutic strategies use drugs like potentiators and correctors or their combination. These drugs act in a mutation-specific manner, making these treatments limited to a few kinds of patients. For this reason, the research is focused on the development of novel classes of drugs: new potentiators and correctors, stabilizers, amplifiers, mRNA repairs and PTC readthrough molecules [1]. Among these, of great interest are the amplifiers that enhance the transcriptional level of *CFTR* gene. Our project is based on the hypothesis that a CF patient with *CFTR* mutations that retain a residual function may have a clinical benefit by increasing the amount of its mutated CFTR protein resulting in an increased chloride ion flux. Thus, our aim is to develop a method for the simultaneous analysis of the effect of molecules on the activity of the *CFTR* gene promoter (through the luciferase assay) [2] and to analyze this effect on the activity of the endogenous CFTR protein through a halide sensitive YFP assay [3].

**Materials and methods**

We constructed a bi-cistronic lentiviral vector (the pCFTR-LUC-YFP) containing two different expression cassettes: the LUC cassette to express the luciferase gene under the control of the CFTR promoter region3kb long; the YFP cassette expressing the yellow fluorescent protein and the puromicyn genes for the endogenous CFTR activity analysis and cell selection. To test our *CFTR* promoter region in as many cell lines as possible, we used pCFTR-LUC-YFP vector for the production of lentiviral particles with VSV-G as envelope protein. Then, we transduced different cell lines with different *CFTR* gene expression levels (i.e., HEK293, A549, Calu3 and Caco 2 cell lines). After treatment with sodium butyrate (NaB), an inhibitor of histone deacetylases, we tested gene expression variation by luciferase assay and RT-PCR[4].

**Results**

Preliminary data show, as expected, that the basal CFTR expression levels are different in each cell lines. Moreover, each cell line differently responds to NaB treatment.

**Conclusions**

The preliminary data demonstrate that this model represents a good screening system for molecules with amplifier action, also by high throughput screening methods. Moreover, the use of lentiviral particles allows to test the activity of the *CFTR* promoter in almost all cell lines as well as in primary cells.

**References**

1. Schmidt BZ, Haaf JB, Leal T et al. Cystic fibrosis transmembrane conductance regulator modulators in cystic fibrosis: current perspectives. Clin Pharmacol 2016;8:127-140

2. Liguori R, Quaranta S, Di Fiore R et al. A novel polymorphism in the PAI-1 gene promoter enhances gene expression. A novel pro-thrombotic risk factor? Thromb Res 2014; 134:1229–33

3. Amato F, Scudieri P, Tomati V et al. Two CFTR mutations within codon 970 differently impact on the chloride channel functionality. Hum Mutat 2019;40:742-8

4. Canani RB, Terrin G, Elce A, et al. Genotype-dependency of butyrate efficacy in children with congenital chloride diarrhea. Orphanet J Rare Dis 2013;8:194

## GI / nutrition

### A13 Evaluation of nutritional status in Cystic Fibrosis patients with end stage lung disease requiring lung transplantation

#### Valeria Daccò, Anna Bulfamante, Francesca Garbarino, Laura Moroni, Laura Claut, Carla Colombo

##### Cystic Fibrosis Center, IRCCS Ca’ Granda, Ospedale Maggiore Policlinico, University of Milan, Milan, Italy

###### **Correspondence:** Valeria Daccò (valeria.dacco@policlinico.mi.it)

**Background**

It is well known that nutritional status is strongly associated with pulmonary function and survival in Cystic Fibrosis (CF). CF patients with end stage lung disease often present a progressive nutritional decline, due to a higher energy expenditure for the increased work of breathing and the chronic pulmonary infection and also due to a reduced calorie uptake. An aggressive nutritional support should be provided in order to maintain or gain additional weight, especially in candidates to lung transplantation (LTx), as poor nutritional status has been consistently associated with increased pre LTx waitlist mortality.

**Materials and methods**

All the CF patients followed in our Center who received LTx between 2006 and 2018 were included in this retrospective study in order to characterize their nutritional status. Clinical data one year before, at the time of listing and at the time of LTx were collected. Nutritional status was evaluated by BMI (weight (kg)/height (m^2^)). According to WHO, patients with BMI < 18.5 were considered underweight.

**Results**

32 patients (18 females), with a median age of 23.3 years (20.4-25.3) at the beginning of the observation were listed. All of them except one were pancreatic insufficient. The median follow up time was 11.7 months (range 0.5-57.4).Twelve months before to be referral for LTx their median BMI was 19.2 [18.3-20.9], with 9patients in whom BMI was <-1,28 DS. No statistically significant changes in median BMI occurred during the time of observation (wait listing: BMI 18.9 [18.2-20.8], time of LTx: BMI: 18.7 [17.9-20.9]). However this lack of deterioration was obtained by means of progressive increase of nutritional intervention (Table 1). A nutritional support (oral nutritional supplements and/or enteral tube feeding) had been already prescribed one year before listing in 38% of cases, which increased to 56% and 59% at the time of listing and at the time of transplantation respectively. In addition a more aggressive nutritional intervention (partial parenteral nutrition) was required in 19% of cases

**Conclusions**

In our patients, BMI was maintained during the waiting list by means of a progressive intensification of nutritional interventions. These findings confirm that therapeutic strategies aimed to maintain weight loss are associated to improve survival in CF patients in waiting list.

An informed consent was obtained from all patients for data publication.

**Table 1 (abstract A13).** Nutrition interventions in patients with end stage lung disease 12 months before, at time of referral for LTx and at time of LTx

	12 months before wait listing	Wait listing	Time of LTx
**Supplemented Patients (%)**	38	56	59
Oral supplementation (%)	31	41	44
Enteral tube feeding (%)	9	13	13
Parenteral supplementation %	0	19	25
Kcal from supplementation % (mean±DS)	12.0 ±18.8	42.0 ±29.1	44.6 ±33.4

No deaths were registered in patients awaiting LTx

### A14 The role of Bioelectrical Impedance in Cystic Fibrosis: the experience of a care Center

#### Giuseppe Scidà, Giuseppina Napoletano, Maria Teresa Arnone, Serena Spigno, Antonio Di Pasqua, Antonella Tosco, Valeria Raia, Anna Coruzzo

##### Cystic Fibrosis Center, Department of Translational Medical Sciences, University Federico II of Naples, Naples, Italy

###### **Correspondence:** Giuseppe Scidà (giuseppe_scida@alice.it)

**Background**

Malnutrition is a common complication in patients with Cystic Fibrosis (CF), and it directly affects the prognosis. Body composition (BC) should be evaluated to identify children at risk of malnutrition. Bioelectrical impedance (BIA) is extensively used to estimate BC. In particular BIA-derived phase angle (PhA) should be used to assess and monitor nutritional status.

**Materials and methods**

From enrolled patients we collected data including CFTR mutation, pancreatic insufficiency status (defined as fecal elastase value < 200 μg/g stool), anthropometric measurement (body weight, height and BMI z-score with the relative percentile), BIA PhA value (φ) performed by HUMAN-iM TOUCH, FEV1% as marker of lung function, liver disorders and glucose tolerance abnormalities.

**Results**

32 CF patients were consecutively enrolled (17 male; mean age M±DS 13.49±3.94 years; range 6.2-17.9 years). 91% were pancreatic insufficient, 16% had severe liver disease, defined according to international recommendations and 59% alterations of glucose metabolism. This cohort had a mean z-BMI -0.075±1.16 and a mean BIA PhA 5.8±0.98 φ (range 4.3-7.9 φ), with a mean FEV1% 91.3±12% (range 40.0%-138.8%). A significant correlation between BIA-PhA and FEV1 (p< 0.05 r=0.58) and z-BMI and FEV1 (p<0.05 r=0.53) was registered. No statistical difference of BIA PhA value was found in CF patients with or without liver disease and in patients with or without alterations of glucose metabolism.

**Conclusions**

Nutritional status strongly affects pulmonary function and survival in CF patients. In our preliminary study BIA PhA seems to have a good relationship with FEV1 values. BIA could be an early tool for nutritional evaluation in children with CF. More data are needed to establish normal values in this population.

All parents gave informed consent to data publication.

### A15 MyCyFAPP: a novel approach for the self management of pancreatic insufficiency in cystic fibrosis

#### Anna MC Bulfamante^1^, Lauretta Valmarana^2^, Rossella Valmarana^2^, Arianna Giana^1^, Rita M Nobili^1^, Carla Colombo^1,3^

##### ^1^Cystic Fibrosis Regional Center, IRCCS Ca’ Granda Foundation Ospedale Maggiore Policlinico, Milan, Italy; ^2^Department of Clinical and Community Sciences, University of Milan, Milan, Italy; ^3^Department of Medical-Surgical Physiopathology and Transplantation, University of Milan, Milan, Italy

###### **Correspondence:** Anna MC Bulfamante (anna.bulfamante@policlinico.mi.it)

**Background**

The MyCyFAPP project was developed to promote self-management of pancreatic enzyme replacement therapy (PERT) in children with Cystic Fibrosis (CF) by means of a mobile application (app) that allows for a personalized and accurate control of pancreatic insufficiency and nutritional status.

**Materials and methods**

The initiative, coordinated by Hospital Universitario La Fe in Valencia, was financed by the European Commission under the Framework Program for Research and Innovation Horizon 2020 and involved CF centers, universities, patient representatives and ITC companies from Spain, Italy, Norway, Germany, Portugal, Belgium and the Netherlands.

**Results**

In the first stage 210 CF patients (30 from the Milan CF Center) completed a four days food record in order to characterize their nutritional habits and deliver common nutritional recommendations. At the same time in vitro digestion studies were carried out to estimate fat digestibility in real foods and meals under CF gastrointestinal conditions (intestinal pH, bile acid concentrations). Subsequently a pilot study was aimed at obtaining a personalized correction factor for each individual on the basis of the Theoretical Optimal Doses (TODs) obtained by in vitro studies. The study consisted of two consecutive stages, throughout which 42 patients (5 from our Center) received a standardized diet and a fixed dose of enzymes. Faecal samples were collected to assess the coefficient of fat absorption (CFA) while patients assumed the TODs. The use of this dose without any correction allowed to obtain a satisfactory CFA. The app was then developed and included an algorithm to calculate PERT-dose, a symptoms diary, a nutritional handbook and educational games. The app was linked to a professional web tool allowing healthcare professionals to evaluate patient’s data and give feedback. A clinical trial was planned to assess its usefulness; in the first stage a questionnaire specifically targeted to gastrointestinal symptoms (PEDSQL-GI) was validated in different languages in 240 CF patients (62 from our Center) to be used as endpoint in the second part of the trial. Cystic Fibrosis Questionnaire-Revised (CFQ-R) and a Visual Analogue Scale (VAS) were also administered. A 6 months multicenter prospective trial involved 154 patients (20 from our Center) who used the app every day entering the meals and taking the PERT dosage calculated. In a substudy of the clinical trial, faecal samples were collected to control CFA on a free diet.

**Conclusions**

The results show that MyCyFAPP improved GI QOL during a 6 months trial and may help patients to improve self-management of PERT.

### A16 Night blindness in Cystic Fibrosis: the central role of vitamin A

#### Laura Zazzeron^1^, Lorenzo Norsa^2^, Laura Claut^1^, Valeria Dacco^1^, Lauretta Valmarana^1^, Anna MC Bulfamante^1^, Arianna Biffi^1^, Carla Colombo^1,3^

##### ^1^Cystic Fibrosis Regional Center, Fondazione IRCCS Ca’ Granda Ospedale Maggiore Policlinico, Milan, Italy; ^2^Pediatric Hepatology, Gastroenterology and Transplantation, ASST Papa Giovanni XXIII, Bergamo, Italy; ^3^Department of Medical-Surgical Physiopathology and Transplantation, University of Milan, Milan, Italy

###### **Correspondence:** Laura Zazzeron (laura.zazzeron@policlinico.mi.it)

**Background**

In children with CF and pancreatic insufficiency, pancreatic enzymes and bicarbonate cannot be secreted into the duodenum, leading to impaired digestion of nutrients, bile acids precipitation, fat and liposoluble vitamins malabsorption. Deficiency of Vitamin A may cause ocular impairment.

**Case Report**

We present the case of a 9 years-old girl with CF diagnosed at birth following intestinal occlusion for meconium ileus (genotype: F508del/L1077P). In the first 6 months of life she underwent two surgeries for occlusion with bowel resection. After surgeries she ended up with 102 cm small bowel, without ileo-ceacal valve, and ileocolic anastomosis. This anatomical situation is classified as short bowel syndrome type 2 and she needed parenteral nutrition (PN) support until the age of 14 months when PN was stopped. Despite the presence of a mild alteration of liver enzymes and a moderate degree of intestinal malabsorption, she had optimal weight and height gain. At the age of 4 years, during a hospital admission for respiratory tract infection, a severe vitamin A deficiency (VitA: 0 mg/l) was documented. This deficiency was associated with nocturnal blindness. Electroretinography (ERG) was performed and found normal. Despite nutritional adaptation of lipid and liposoluble oral intake, the deficit could not be corrected and was associated with important malnutrition which justified a new PN support. This was continued for 18 months with improvement of nutritional status and liposoluble vitamin profile (VitA:0,16mg/l, VitE:6,7mg/l, VitD:27,6 mcg/l). PN was stopped because of hepatic deterioration with cholestasis and evolution to cirrhosis. Two years after interruption of PN, despite normal growth and no clinical symptoms, retinal sufferance was found in a follow-up ERG. Vitamin A level was 0,11 mg/l with the rest of lipid soluble vitamins in range. After an unsuccessful increase of oral supplementation, intramuscular administration was started leading to ERG normalization.

**Conclusion**

The peculiarity of this case is the coexistence of alteration in the three main protagonists of Vitamin A metabolism: pancreas, bowel and liver. Those three organs have a strong anatomic and functional linkage as testified by “the vitamin A vicious circle” [1].

The informed consent of the parents has been obtained.

**Reference**

1. Wiseman EM, Bar-El Dadon S, Reifen R. The vicious cycle of vitamin a deficiency: A review. Crit Rev Food Sci Nutr 2017;57:3703–14

### A17 Evaluation of the adherence of NaCl supplementation with Wadi (cps NaCl 1g + Mg) in CF

#### Benedetta Fabrizzi, Andrea Benigni, Veronica Zamponi, Marco Cipolli

##### Cystic Fibrosis Referral Care Center, Mother-Child Department, Ospedali Riuniti, Ancona, Italy

###### **Correspondence:** Benedetta Fabrizzi (benedetta.fabrizzi@ospedaliriuniti.marche.it)

**Background**

Sodium supplementation (as sodium chloride) is essential for patients with Cystic Fibrosis, however adherence to the nutritional therapy is often below the optimal value [1]. The are several causes: from the very low palatability of the products used to the huge daily therapeutic load of CF patients that leads nutritional supplementation therapy to take second place.

**Materials and methods**

Collection of data regarding drug withdrawals in terms of quantity and frequency at local patients’ pharmacies by telephone interview and / or email and comparison with the medical prescription.

**Results**

78% of patients shows adherence to sodium supplementation with Wadi (NaCl 1 g + Mg capsule).

**Conclusions**

Adherence to sodium supplementation with Wadi is very close to the optimal value of 80%.The limit of the study is that the withdrawal of the drug does not necessarily imply its intake. Although this drug has recently come on the market, the data of its use are encouraging, as it presents a neutral palatability and a format that meets the patient's habits.

**Reference**

1. Narayanana S, Mainz JG, Gala S, et al. Adherence to therapies in cystic fibrosis: a targeted literature review. Expert Rev Respir Med 2017; 11:129-45.

## Infection / inflammation

### A18 Non Tubercolous Mycobacteria (NTM) in cystic fibrosis (CF) patients: real-life data from Verona CF center

#### Giulia Cucchetto^1^, Francesca Lucca^2^, Emily Pintani^1^, Marisol Ocampo Barao^1^, Sonia Volpi^1^

##### ^1^Cystic Fibrosis Regional center, Ospedale Civile Maggiore, Verona, Italy; ^2^Pediatrics School, University of Verona, Verona, Italy

###### **Correspondence:** Giulia Cucchetto (giulia.cucchetto@yahoo.it)

**Background**

NTM are one of the most challenging pathogens in patients with CF. Diagnosis and specific treatments can be difficult in the clinical practice despite guidelines have been published. The aim of our study is to collect clinical and microbiological data of CF patients with NTM finding followed at our center.

**Materials and methods**

Data about CF patients with airway NTM isolation from June 2006 to June 2019 were extracted from our database (with prospectic insertion).

**Results**

Airway NTM was reported in 41 patients, representing 5.1% of the whole CF population followed at our center. Females represented 51.22% of our population; delF508 was in homozygosis in 19.51%, and heterozygosis in 56.1%; 73.17% was pancreatic insufficient, while 34.15% had CFRD. Chronic P. aeruginosa, MSSA, MRSA colonization had a prevalence of 82.93%, 36.58%, 12.19% respectively; Aspergillus was found in 61% of patients; S. apiospermum was isolated in 3 patients. First NTM finding resulted at 29.05 ±14.27 years old. M. abscessus, M. intracellulare and M. avium spp were isolated in 46.34%, 24.39% and 9.76% respectively. In the transplanted patients included, NTM was isolated in 2 patients after transplantation, in 5 patients before transplantation. Mean FEV1 in the 12 months before NTM finding was 68.45 ±24.47 %pred, mean FEF25-75 in the 12 months preceding NTM was 46.80 ±49.37 %pred; no change was shown in the 12 months following the first NTM finding. Treatment for NTM was performed in 17 patients (41.46%). The therapy is still ongoing in 5 patients, in 4 of them sputum culture for NTM is still positive (all of them with M. abscessus). Twelve patients concluded the treatment after a minimum duration of 12 months, within this group NTM is still present in 4 patients (all of them with M. abscessus) and eradicated in 8 patients (50% with M. abscessus). Admission to hospital decreased in the 12 months following NTM finding in comparison to 12 months preceding NTM, but this was significant only in the subset of patients treated for NTM.

**Conclusions**

NTM prevalence complies to literature. Even if therapy is still ongoing in some patients, NTM was eradicated only in 52.94%, proving the challenging nature of NTM treatment. This appears more evident for M. abscessus than the other species. Fewer hospital admissions in the treated population may suggest a control over infection and inflammation associated to treatment. Assessing treatment response and clinical data in the different NTM species requires wider populations and further studies.

### A19 Early Pseudomonas Aeruginosa (PA) identification in 2003-2013 birth cohort cystic fibrosis (CF) patients: clinical, functional and radiological impact

#### Francesca Lucca^1^, Giulia Paiola^2^, Emily Pintani^2^, SoniaVolpi^2^

##### ^1^Pediatrics School, University of Verona, Verona, Italy; ^2^Cystic FibrosisRegional center, Ospedale Civile Maggiore, Verona, Italy

###### **Correspondence:** Francesca Lucca (francesca.lucca@hotmail.it)

**Background**

Since early events in CF patients’ life can determine disease progression and burden in later years, there is increasing interest regarding these events. The aim of our study was to evaluate the effect of PA first identification in preschoolers.

**Materials and methods**

We retrospectively evaluated data regarding 150 2003-2013 birth cohort CF patients with regular follow-up. All patients had chest CT performed at 4-7 years of age, forced spirometry at 6 years and first N2-MBW measurement.

**Results**

We identified 3 groups: 61 patients with first PA identification before 2 years of age (group A); 47 patients with first PA at 2-6 years of age (group B); 42 patients without PA up until 7 years of age(group C). Class I or II mutations on both alleles were significantly more frequent in groups A and B (79% and 72.3% vs 36%), with an OR of 5.98 for PA identification before 7 years of age. Pancreatic insufficiency was significantly more frequent in groups A and B than in group C (91.8% e 85.1% vs 40.48%), with an OR of 11.76 for PA identification before 7 years of age. BMI percentile was higher in group C (p<0.01). FEV_1_%pred was worse in group A than in group C (98.66±15.38 vs 107.19±17.35, p=0.012), as was FVC (p=0.029). LCI 2.5% was worse in groups A and B than in group C (9.45 [8.93-12.6] and 10.82 [8.47-13.52] vs 7.91 [7.24-11.13], p= 0.0023); analysis of LCI 2.5%pred was consistent with absolute values; S_cond_was also significantly worse in groups A and B; S_acin_was no different between groups. The prevalence of normal lung CT was lower in group A (19,67%, p=0.0006) and B (22,4%, p=0.005), than C (52.38%); PA identification before 2 and 7 years of age associates with an OR of abnormal chest CT of 2.4 and 4.06, respectively. Peribronchial thickness and mucus plugs were significantly more frequent in groups A and B than in group C. No significant difference was shown between groups A and B for all examined parameters. Chronic colonization developed in 19.67% of group A and in 21.28% of group B. MRSA was found in 3.3% of A, 8.5% of B, 4.8% of C.

**Conclusions**

Of note, early PA isolation impacts on respiratory function and radiological status in CF preschool children. Nonetheless, chronic colonization only develops in a relatively small subgroup.

### A20 Longitudinal genomic analysis of *Pseudomonas aeruginosa* as a tool for the definition of persistence/reinfection in the airways of Cystic Fibrosis patients

#### Ramona Pezzotta^1^, Piercarlo Poli^2^, Chiara Bracchi^3^, Sara Roversi^1^, Alberto Zani^1^, Erika Scaltriti^1,3^, Rita Padoan^2^, Arnaldo Caruso^1^, Simona Fiorentini^1^

##### ^1^Department of Molecular and Translational Medicine, Section of Microbiology, University of Brescia/ASST Spedali Civili, Brescia, Italy; ^2^Department of Pediatrics, Cystic Fibrosis Support Center, Children’s Hospital, ASST Spedali Civili, Brescia, Italy; ^3^Risk Analysis and Genomic Epidemiology Unit, Istituto Zooprofilattico Sperimentale della Lombardia e dell'Emilia-Romagna, Parma, Italy

###### **Correspondence:** Ramona Pezzotta (ramypezzotta@gmail.com)

**Background**

In cystic fibrosis (CF) patients chronic *Pseudomonas aeruginosa* (*Pa*) infection is associated with lung damage, a more rapid decline in lung function, and is an important prognostic factor of morbidity and mortality [1]. *Pa* earlier acquisition shortens life expectancy, therefore, eradication of initial *Pa* acquisition and delay chronic infection is crucial for patient care [2]. Aim of this study was to analyse the whole genome sequences (WGS) of *Pa* isolates obtained from a child over a 4 years period in order to define if she was subjected to uncommonly frequent reinfections or if she has acquired an early chronic *Pa* infection.

**Materials and methods**

*Pa*isolates (n = 32) were subjected to WGS using the Nextera XT Flex DNA kit and the Miseq system (Illumina). Genetic characterization was performed by comparison of the obtained *Pa* genome sequences with specific databases such as virulence gene and antibiotic resistance databases. Moreover, phylogenetic relationship of the isolates was evaluated using a SNP-based approaches and SNPs matrix was used as input for phylogenetic analyses to determine the relationships between genomic sequences of *Pa* isolates obtained from this study and the ones that are present in databases.

**Results**

WGS analysis, using PAO1 as reference genome of *Pa*, highlighted the presence of two clusters whose isolates differ in about 1000 pairwise SNPs. Within the same cluster, *Pa* isolates had a maximum of 6 SNPs difference confirming the clonality of different isolates. The main cluster comprises all the*Pa* strains isolated in the period 2015-2017, when the child had two >6-months period of *Pa*-free cultures and some strains isolated in 2018/2019 (cluster I), whereas the cluster II contains only recent strains (years 2018-2019).

**Conclusions**

Results have shown that, starting the first *Pa* isolation, the child suffered from a chronic infection and that a superinfection occurred some years later. Evaluation of *Pa* clonality by WGS may support studies aimed to determine efficacy of eradication therapies and may help to manage patients for obtaining a better clinical outcomes.

**References**

1. Emerson J, Rosenfeld M, McNamara S et al. Pseudomonas aeruginosa and other predictors of mortality and morbidity in young children with cystic fibrosis. Pediatr Pulmonol 2002;34:91-100

2. Marchetti F, Giglio L, Candusso M, et al. Early antibiotic treatment of pseudomonas aeruginosa colonisation in cystic fibrosis: a critical review of the literature. Eur J Clin Pharmacol 2004; 60:67-74

### A21 Protective role of Palivizumab in the prevention of RSV respiratory infections in patients with Cystic fibrosis

#### Francesca Ficili, Annalisa Ferlisi, Maria Antonietta Orlando, Lisa Termini, Gabriella Traverso, Maria Rita Bonaccorso, Maria Antonietta Calamia, Mirella Collura

##### Cystic Fibrosis Center and Chronic Respiratory Diseases, O.U. Second Pediatrics “Di Cristina Hospital”, ARNAS CIVICO, Palermo, Italy

###### **Correspondence:** Francesca Ficili (fficili@hotmail.com)

**Background**

Respiratory syncytial virus (RSV) is one of the most important pathogenic viruses with airway tropism. It is responsible for various clinical manifestations including bronchitis, pneumonia and bronchiolitis. Severe forms of bronchiolitis can affect pediatric patients, especially patients suffering from chronic respiratory diseases including cystic fibrosis. To date, active immunization is not yet available, but there is the possibility of carrying out passive immunization through the administration of monoclonal antibodies (Palivizumab). Few studies have evaluated protective efficacy of these antibodies especially in pediatric patients with cystic fibrosis (CF).

**Materials and methods**

We performed a retrospective analysis (2014-2019) involving all CF patients in follow-up in our Unit that presented respiratory symptoms with need of hospitalization, to evaluate the incidence of RSV related infections in pediatric patients younger than 24 months who had received prophylactic treatment with Palivizumab. All patients underwent RSV research in nasal mucus. All patients received five doses of Palivizumab in the first year of life.

**Results**

We identified 37 patients (22F e 15M) with CF born between 2014 and 2019 with diagnosis by screening or by symptoms, carried out within 2 years of age, who received prophylaxis with Palivizumab in the first year of life (five doses 15mg/kg/dose). 3 patients (8.1%) experienced RSV infection during hospitalization for respiratory problems, two of them with other comorbidities (extreme prematurity and bowel obstruction) and only one without other risk factors. 34 patients (91.9%) never had RSV infection.

**Conclusions**

Since there are no absolute indications about the administration of Palivizumab in CF patients, our analysis showed that this passive prophylaxis led to a low incidence of RSV infections. It therefore seems reasonable to implement a prophylaxis program with Palivizumab in patients suffering from chronic respiratory diseases such as CF.

### A22 Potential role of serum and plasmatic biomarkers to predict clinical and functional response to antibiotic treatment for pulmonary exacerbation

#### Benedetta Fabrizzi^1^, Chiara Gelardi^1^, Valentino Bezzerri^1^, Marisole Allegri^1^, Lidia Da Lio^2^, Barbara Cinti^2^, Marco D’Anzeo ^2^, Luisita Marinelli^2^, Valentina Iencenella^3^, Silvia Staffolani^3^, Gloria Tridello^4^, Anastasia D’Antuono^1^, Federica Masseria^1^, Giuseppe Scopelliti^1^, Eleonora Cesaretti^1^, Marco Cipolli^1^

##### ^1^Cystic Fibrosis Regional Referral Centre, Maternal-Infant Department, Ospedali Riuniti, Ancona, Italy; ^2^Laboratory Analysis, Services Department, Ospedali Riuniti, Ancona, Italy; ^3^Unit of Emerging and Immunosuppressed Infectious Diseases, Department of Gastroenterology and Transplantation, Ospedali Riuniti, Ancona, Italy; ^4^Cystic Fibrosis Centre, Azienda Ospedaliera Universitaria Integrata, Verona, Italy

###### **Correspondence:** Benedetta Fabrizzi (benedetta.fabrizzi@ospedaliriuniti.marche.it)

**Background**

Pulmonary exacerbations (PEx) frequently occur in cystic fibrosis patients, with an unfavorable impact on disease course [1]. The need of precocious markers of PEx as diagnostic tools and prognostic factors for treatment response, has increased the interest to biomarkers analysis in both sputum and blood samples. Several studies have investigated the role of acute phase reactants during PEx and little evidence exists for serum C-reactive protein (CRP) [2] and sputum interleukin-8 (IL8) [3]. In this study we measured several plasmatic and serum biomarkers to estimate their predictive and prognostic value in course of PEx.

**Materials and methods**

We prospective enrolled 24 CF patients (17 F, 7 M) in course of PEx in need of intravenous antibiotic treatment. Patients were assessed at the diagnosis of PEx, at the fifth day of treatment and at the end of the antibiotic course. During each control patients performed clinical evaluation, pulmonary function test (forced expiratory volume in one second, FEV1) and blood test of plasmatic and serum biomarkers (fibrinogen, FBG; calprotectin, CP; interleukin-6, IL6; IL8; procalcitonin, PCT; white blood cells, WBC; reticulocyte, RCT; erythrocytes sedimentation rate, ESR; CRP). Informed consent was obtained from all patients for data publication.

**Results**

The main results of our study are:

1. High levels of at least two serum biomarkers at the diagnosis of PEx occurred in all but two patients;
2. All patient completed the antibiotic treatment (14 days) and we observed a significant reduction of IL6, CRP, CP, FBG and WBC at the end of the antibiotic course (Table 1), despite this reduction was independent with favorable and unfavorable response;
3. FBG seems to be the most earlier and predictive marker of treatment efficacy: the reduction of FBG after five days of therapy was associated to significant improvement in FEV1 values at the end of the antibiotic cycle.

**Conclusions**

Serum and plasmatic biomarkers seems to be simple and reproducible parameters to assess clinical and functional response during PEx and the trend of FBG may reflect the improvement of lung function. Further studies, with a large population, are needed to better investigate the role of such markers in clinical practice.

**References**

1. Bilton D, Canny G, Conway S et al. Pulmonary exacerbation: towards a definition for use in clinical trials. Report from the EuroCareCF working group on outcome parameters in clinical trials. J Cyst Fibros 2011; 10 Suppl 2:S79-81

2. Sagel SD, Thompson V, Chmiel JF et al. Effect of treatment of cystic fibrosis pulmonary exacerbation on systemic inflammation. Ann Am Thorac Soc 2015; 12:708-17

3. Breuer O, Caudri D, Stick S and Turkovic L. Predicting disease progression in cystic fibrosis. Expert Rev Respir Med 2018; 12:905-917

**Table 1 (abstract A22).** Biomarkers trend during antibiotic course (T0 – T14)

Biomarkers	Range	Mean (SD)	Median	p-value
Fibrinogen (mg/dl)	-169.0 - 534.0	126.41 (149.78)	86.0	p <.0001
Calprotectin (ug/ml)	-4.9 - 55.8	6.80 (14.60)	1.7	p=0,0092
White blood cells (10^3^/mmc)	-2490.0 - 6220.0	2079.09 (2786.60)	1870.0	p=0,0022
Procalcitonin (ng/ml)	-0.9 - 0.1	-0.03 (0.19)	0.0	p=0,3534
Interleukin 6 (pg/ml)	-9.1 - 155.5	16.34 (39.45)	2.2	p=0,0165
Interleukin 8 (pg/ml)	-506.8 - 3737.0	172.86 (826.87)	-2.1	p=0,6995
Erythrocytes sedimentation rate (mm/h)	-4.0 - 50.0	15.83 (18.21)	11.5	p=0,0073
Reticulocyte (mmc)	-90000.0 - 32600.0	-16172.22 (30994.7)	-12550.0	p=0,0483
C reactive protein (mg/dl)	-0.2 - 7.3	1.26 (2.12)	0.3	p=0,0002

### A23 Salivary cytokines and lung disease evaluation in patients with Cystic Fibrosis

#### Alice Castaldo^1^, Renato Liguori ^2^, Paola Iacotucci^3^, Vincenzo Carnovale^3^, Serena Buonaurio^3^, Chiara Cimbalo^4^, Antonella Tosco^4^, Valeria Raia^4^, Monica Gelzo^5^

##### ^1^Department of Public Health, University of Naples “Federico II”, Naples, Italy; ^2^CEINGE- Advanced Biotechnology, Naples, Italy; ^3^Cystic Fibrosis Center, Adult Unit, Department of Translational Medical Sciences University of Naples “Federico II”, Naples, Italy; ^4^Cystic Fibrosis Center, Pediatric Unit, Department of Translational Medical Sciences University of Naples “Federico II”, Naples, Italy; ^5^Department of Molecular Medicine and Biotechnology, University of Naples Federico II, Naples, Italy; CEINGE- Advanced Biotechnology, Naples, Italy

###### **Correspondence:** Alice Castaldo (ali.castaldo@hotmail.it)

**Background**

In order to detect infections and quantify inflammatory biomarkers in patients with CF, bronchoalveolar lavage fluid (BALF) and/or sputum has been previously performed with conflicting results on their use. Saliva could represent an useful alternative tool being characterized by non-invasive collection and direct anatomical relationship with the lower airways [1]. The aim of this study was to investigate whether the salivary levels of interleukin-6 (IL-6), IL-8 and tumour necrosis factor-alpha (TNF-α) are associated with the clinical status of CF patients.

**Materials and methods**

Unstimulated saliva samples from 110 CF adults and 32 CF children have been collected at the Regional Cystic Fibrosis Center, Adult and Pediatric Section. and from 50 healthy subjects as controls. Lung disease severity was classified as severe, moderate and mild considering both the FEV_1_ and age of patients [2]. Salivary biochemical parameters were analyzed by automated clinical chemistry analyzer. Salivary IL-6, IL-8 and TNF-α were measured by specific ELISA methods.

**Results**

Biochemical analyses revealed that salivary chloride was significantly higher (p<0.05) in CF adults compared to controls, while calcium and phosphate resulted lower (p<0.005). Furthermore, salivary LDH, potassium and phosphate concentrations were significantly higher in CF children compared to those in CF adults (p<0.05). All three salivary cytokines, IL-6, IL-8 and TNF-α, resulted significantly higher in CF adults compared to controls (Table 1). No significant differences were observed between CF adults and children. Regarding the correlations between cytokines and the lung disease severity in CF patients significant correlations were observed only in CF children: i) Spearman’s rank-order analysis showed a positive significant correlations between IL-8 and FEV_1_ (r_s_: 0.388; p=0.031); ; ii) IL-6 positively correlated with FEV_1_/age ratio, an index of lung disease severity (r_s_: 0.412; p=0.019). No significant correlations of the salivary cytokines levels with the genotype and lung colonization were observed in neither CF adults and children.

**Conclusions**

This study showed that:

- salivary electrolyte and LDH concentrations were significantly different among healthy subjects, CF adults and children;
- IL-6, IL-8 TNF-α levels were significantly higher in saliva from CF patients compared to healthy subjects;
- in CF children, IL-6 and IL-8 correlated positively and negatively, respectively, with lung disease severity.

According with the literature [3], our results suggest that saliva could represent a valid matrix for the diagnosis and monitoring of CF patients. In particular, salivary IL-6 and IL-8 could represent useful biomarkers for monitoring lung disease in CF children. Further studies are needed to confirm the power of salivary markers and to define their potential predictive value.

Informed consent to data publication was obtained from all parents.

**References**

1. Nunes LA, Mussavira S, Bindhu OS. Clinical and diagnostic utility of saliva as a non-invasive diagnostic fluid: a systematic review. Biochem Med 2015;25:177-92

2. Schluchter MD, Konstan MW, Drumm ML, et al. Classifying severity of cystic fibrosis lung disease using longitudinal pulmonary function data. Am J Respir Crit Care Med 2006;174:780-6

3. Gonçalves AC, Marson FA, Mendoca RM et al. Saliva as a potential tool for cystic fibrosis diagnosis. Diagn Pathol 2013;8:46

**Table 1 (abstract A23).** Cytokines levels in controls subjects, adult and children CF patients as median (2.5-97.5 %ile)

Cytokines (pg/mL)	Controls (n = 50)	CF adults (n = 70)	CF children (n = 31)	Kruskal-Wallis (p value)
IL-6	28.2 (4.1-66.8)	57.9 (18.3-202) ^a^	57.6 (17.7-357)	5.15*10^-10^
IL-8	55.0 (11.8-206)	307 (55.3-1031) ^a^	245 (33.4-889)	1.06*10^-13^
TNF-α	12.4 (0.5-56.1)	27.4 (2.4-94.1) ^a^	30.8 (12.6-87.2)	2.16*10^-6^

^a^ CF adults versus controls, p < 0.00001

## Respiratory

### A24 Therapeutic bronchoscopy with administration of recombinant human deoxyribonuclease in patients with cystic fibrosis

#### Giuseppe Fabio Parisi, Maria Papale, Sara Manti, Enza Mulé, Donatella Aloisio, Novella Rotolo, Salvatore Leonardi

##### Cystic Fibrosis Center Catania, AOU Policlinico Vittorio Emanuele, University of Catania, Catania, Italy

###### **Correspondence:** Giuseppe Fabio Parisi (giuseppeparisi88@hotmail.it)

**Background**

Cystic fibrosis is characterized by a progressive respiratory disease that is still the leading cause of morbidity and mortality. The accumulation of mucus with consequent neutrophil-mediated inflammation is responsible for the formation of bronchiectasis, which feeds the infectious-inflammatory vicious circle. Furthermore, during inflammation, there is a release of deoxyribonucleic acid (DNA) which contributes to increasing the density of secretions. To interrupt this vicious circle, in recent years, the method of bronchoscopy with therapeutic lavage has been taking hold with the instillation of recombinant human DNase to make the secretions more fluid and then aspirating them avoiding their accumulation and reducing inflammation [1].

**Materials and methods**

Herein we describe our experience using bronchoscopy with instillation of recombinant human deoxyribonuclease (rhDNase) in five adults with CF regardless the evidence of lobar atelectasis. RhDNase (2.500 U/2.5ml diluted in 20 mL 0.9% saline) was administered into the most affected lobes (identified by chest radiograph prior to the procedure) by flexible bronchoscopy.

Patients data were the following:

- 36 year-old man, genotype F508del/R553X, chronic *Pseudomonas aeruginosa* colonization with dyspnea and increased sputum production. Chest computed tomography (CT) revealed areas of mucus plugging above all in the right middle lobe;
- 28 year-old woman, genotype F508del/F508del in treatment with Lumacaftor/Ivacaftor, chronic *Klebsiella pneumoniae* colonization with recent impairment in lung function. Chest X-ray showed consolidation of right middle and lower lobes;
- 19 year-old male, genotype F508del/W1282W, chronic methicillin-resistant *Staphylococcus aureus* and areas of consolidation on chest X-ray in the right middle and lower lobes;
- 18 year-old male, genotype F508del/G542X, allergic bronchopulmonary aspergillosis in treatment with Omalizumab and atelectasis of the right upper lobe;
- 38 year-old male, genotype F508del/F508del in treatment with Lumacaftor/Ivacaftor, chronic *Pseudomonas aeruginosa* colonization and recent worsening of respiratory symptoms and lung function. Chest X-ray showed persistent consolidation of the left lower lobe.

**Results**

In four out of the five cases, the procedure resulted in an immediate improvement in symptoms, forced expiratory volume in 1 s (FEV1) and radiological features. Following the procedure, the patients resumed their regular medical regimen which included nebulised rhDNase.

**Conclusions**

In our hands, bronchoscopic instillation of rhDNase in patients with CF was safe and well-tolerated. These preliminary observations are encouraging. However, randomized controlled prospective trials of bronchoscopic instillation of rhDNase are needed to determine whether this form of treatment is justified.

All patients gave written informed consent for data publication.

**Reference**

1. Paul L. Is bronchoscopy an obsolete tool in cystic fibrosis? The role of bronchoscopy in cystic fibrosis and its clinical use. J Thorac Dis. 2017 Sep;9(Suppl 10):S1139-S1145. doi: 10.21037/jtd.2017.06.143.

### A25 Inhaled dry powder mannitol tolerability in cystic fibrosis (CF) patients is influenced by respiratory function and age

#### Giulia Paiola^1^, Francesca Lucca^2^, Marisol Ocampo Barao^1^, Sara Tomezzoli^1^, Antonella Borruso^1^, Laura Menin^1^, Emily Pintani^1^, Sonia Volpi^1^

##### ^1^Cystic Fibrosis Regional center, Ospedale Civile Maggiore, Verona, Italy; ^2^Pediatrics School, University of Verona, Verona, Italy

###### **Correspondence:** Giulia Paiola (giulia.paiola@aovr.veneto.it)

**Background**

Inhaled mannitol dry powder is an osmotic agent which hydrates airway surface liquid and can be prescribed in adult CF patients with FEV1 >30%pred (pp). A recent study showed its safety and tolerability also in patients aged 6-17 years [1]. The aim of our study was to evaluate tolerability in CF patients followed at our Center.

**Materials and methods**

We retrospectively evaluated data regarding patients who underwent mannitol tolerance test (MTT) between March 2017 and June 2019. Clinical and functional data regarding the year before and the period after MTT, were extracted.

**Results**

MTT was performed in 47 patients (F 68.08%), mean age 28.15 (range 11.38-50.51), mean pp FEV1 in the previous 12 months 71.6 ±17.7. MTT was tolerated (and mannitol treatment initiated) in 30 patients (63.83%) [group A], of which 7 were younger than 18; mean fall in ppFEV1 from baseline was 7.38 ±5.46%.17 patients (1 was younger than 18) did not tolerate MTT [group B]; mean fall of ppFEV1 from baseline was 14.1 ±7.44% (significantly higher than in group A); 6 patients presented delta ppFEV1 ≥20%, while 17 patients presented irritative cough. Patients in group B were older , though not significantly (mean age 30.20 ±9.86 vs 26.99 ±9.83); they had also lower mean ppFEV1 in the previous year (64.48 ±15.95 vs 75.7 ±17.57, p=0.03), and lower mean ppFEF25-75 in the previous year (29.1 ±16.75 vs 43.15 ±23.12, p=0.03); no difference was shown in sex and ppFVC.

ppFEV1, FEF25-75 and FVC did not change after MTT in both groups, (considering an “after” period of at least 4 months, with 2 spirometries performed). Among group A, therapy is ongoing in 21 patients (70%) after mean 0.72years. 9 patients discontinued treatment after mean 0.5 years, due to hemoptisis (2 patients), irritative cough (6 patients) and autonomous choice (1 patient); this subset had lower ppFEV1 in the year before MTT than the subset with ongoing therapy (67.76 ±17.61 vs 79.08 ±16.83), though not significantly.

**Conclusions**

MTT is feasible in pediatric and adult patients. MTT was proposed in patients with FEV1> 30% not tolerating hypertonic saline, or requiring an additional mucolytic agent, or a portable and faster mucolytic therapy. In our experience, mannitol therapy coud be initiated in 64% of patients with these characteristics. The MTT positive outcome was shown to be influenced by a better respiratory function and a younger age.

**Reference**

1. De Boeck K, Haarman E, Hull J et al. Inhaled dry powder mannitol in children with cystic fibrosis: a randomized efficacy and safety trial. J Cyst Fibros 2017;16:380-7

### A26 Lung clearance index evaluation in detecting nocturnal hypoxemia in Cystic Fibrosis patients: toward a new diagnostic tool

#### Maria Papale, Giuseppe Fabio Parisi, Sara Manti, Lucia Spicuzza, Enza Mulè, Novella Rotolo, Salvatore Leonardi

##### Cystic Fibrosis Center Catania – AOU Policlinico-Vittorio Emanuele – University of Catania – Catania, Italy

###### **Correspondence:** Maria Papale (mariellapap@yahoo.it)

**Background**

Nocturnal hypoxemia adversely affects outcomes in patients with cystic fibrosis (CF). Although an early detection of this abnormality may be desirable, still its predictability remains uncertain. The Lung Clearance Index is a measure of lung ventilation distribution obtained from a multiple-breath washout technique (MBW), recently implemented in patients with CF. This study aimed to establish whether the LCI predicts nocturnal hypoxemia in patients with stable CF, with mild to moderate disease and normal diurnal gas exchange.

**Materials and methods**

31 stable patients (15 males, mean age 17.4±5.2 years) with mild to moderate CF, normoxic when awake, were enrolled. In all patients we performed nocturnal cardio-respiratory polygraphy, lung function measurement and MBW test to derive LCI values.

**Results**

LCI was abnormal in most of the patients and inversely correlated with mean nocturnal SpO_2_ (r=-0.880 p<0.01). A receiver operating characteristic (ROC) analysis, performed to assess whether LCI predicted nocturnal hypoxemia, revealed a high predictive accuracy of LCI for nocturnal desaturation (AUC=0.96; Youden index=0.79). Forced expiratory volume in 1 second (FEV_1_) was predictive only in patients with more severe airway obstruction, with a moderate degree of accuracy (AUC 0.71) (Table 1).

**Conclusions**

The LCI showed a high effectiveness in predicting nocturnal hypoxemia in stable patients with CF, particularly when compared with a traditional parameter of lung function such as FEV_1_.

**Table 1 (abstract A26).** Comparison of demographic, clinical and polygraphic variables of the patients according to FEV_1_ values

Patients*	FEV_1_<65%	FEV_1_>65%	p value
N.	14	17	
Gender (M:F)	7:7	8:9	n.s.
Age (years)	18.9±4.7	16.1±5.4	n.s.
BMI (<18/>18)	11/3	10/7	n.s.
Pulmonary colonization			
Pa	*9*	*2*	<0.01
Sa	*4*	*10*	<0.05
Bc	*1*	*0*	n.s.
Nbf	*0*	*5*	<0.05
LCI	*17.4*±3.1	*9.6*±3.5	<0.01
FEV1%	*48.2*±12.2	*80.1*±10.8	<0.01
Mean awake SpO_2_	*96%*±0.009	*98%*±0.007	n.s.
Mean Nocturnal SpO_2_	*91%*±0.01	*94%*±0.01	<0.01
Time with SpO_2_<90%	*8%*±0.09	*2.38%*±0.28	<0.01
Mean TcCO_2_ (mmHg)	*32.6*±3.33	*32.6*±3.09	n.s.
ODI	*7.2*±0.7	*7.2*±1.0	n.s.
AHI	*0.5*±0.3	*1.7*±2.1	n.s.
Mean awake RR	*21.3*±2.2	*20*±1.3	n.s.
Mean nocturnal RR	*27.4*±3.8	*21.9*±2.1	<0.01

M: male; F: female; BMI: body mass index; Pa: Pseudomonas aeruginosa; Sa: Staphylococcus aureus; Bc:*Burkholderia cepacia;* Nbf: normal bacterial flora; LCI: lung clearance index; FEV1: forced expiratory volume in 1 s; TcCO_2_: transcutaneous partial pressures of carbon dioxide; ODI: oxygen desaturation index; AHI: Apnea Hypopnea Index; RR: respiratory rate. Data are presented as mean ± standard deviation

### A27 Chronic rhinosinusitis in cystic fibrosis: a review of surgical management and our surgical experience

#### Maurizio Di Cicco^1^, Daniele Di Pasquale^1^, Marco Borin^1^, Giovanna Pizzamiglio^2^, Carla Colombo^2^

##### ^1^ENT Department, Foundation Cà Granda, IRCCS Policlinico Hospital, Via F. Sforza 35, 20122, Milan, Italy; ^2^Regional Referral Centre for Cystic Fibrosis, Foundation Cà Granda, IRCCS Policlinico Hospital, Via F. Sforza 35, 20122; University of Milan, Milan, Italy

###### **Correspondence:** Maurizio Di Cicco (maurimdc@yahoo.com)

**Background**

Almost all the patients with Cystic Fibrosis (CF) present chronic rhinosinusitis (CRS). Clinical and basic scientific research, focusing on therapeutic strategies for CF-associated CRS, is limited; endoscopic sinus surgery (ESS) is an option for patients with CRS, it can help in management of infection, improve quality of life and stabilize lung function decline.

**Material and methods**

Pertinent studies published between January 2015 and January 2019 were selected by a Medline search accessed via PubMed and the Cochrane Library of titles and abstracts using the standard Boolean system; the words “endoscopic sinus surgery AND rhinosinusitis AND cystic fibrosis” were used as search string.

**Results**

Despite appropriate medical therapy, 20-60% of CF patients are going to require ESS; surgical intervention is generally reserved for those who have failed more conservative medical therapy [1]; to reduce pulmonary pathogen colonization, especially in transplant CF patients, is mandatory [2]. Proposed predictive criteria for ESS are: massive polyposis, prior history of ESS, high Lund-Mackay score, high SNOT-22 score and severe CFTR mutations [3,4]; delay in surgery did not affect post-operative improvement [5]. A pre-operative evaluation of CT findings is essential to avoid complications; an intra-operative image guidance can be useful due to anatomic differences in CF patients, especially in revision cases [6]. ESS procedure in pediatric patients is totally safe [7]. ESS also plays a critical role in reducing or eradicating pulmonary colonization of pathogens in CF patients [8]. Surgery leads to relieve nasal obstruction, decrease purulent nasal discharge, increase activity level and improve olfaction [9]. At our ENT-CF clinic, established 1989, CF patients are evaluated with CT and cone-beam CT (CBCT) for pediatric patients; radiological results are qualitatively and quantitatively evaluated using Lund-Mackay score and together with endoscopic Meltzer's Score and SNAQ-11 questionnaire are used for the assessment of a CF sinus score (CFSS). In the last 5 years, 88 patients (51.1% under 18 years old) underwent ESS with clinical and symptomatic improvement. Our experience is consistent with the results in the literature regarding similar strategies.

**Conclusions**

The treatment of CRS in CF is complex and challenging; currently available data are limited to mostly case series and further larger perspective studies are much needed. ESS has been shown to improve sinus and pulmonary bacterial colonization, as well as reducing patient symptoms. Increasing research suggested that a multi-disciplinary approach with ESS combined with topical and medical therapies offer the most optimal treatment for CF patients.

**References**

1. Tipirneni KE, Woodworth BA, Medical and Surgical Advancements in the Management of Cystic Fibrosis Chronic Rhinosinusitis. Curr Otorhinolaryngol Rep 2017;5:24-34

2. Choi KJ, Cheng TZ, Honeybrook AL, et al. Correlation between sinus and lung cultures in lung transplant patients with cystic fibrosis. Int Forum Allergy Rhinol 2018;8:389-93

3. Ayoub N, Thamboo A, Habib AR et al. Determinants and outcomes of upfront surgery versus medical therapy for chronic rhinosinusitis in cystic fibrosis. Int Forum Allergy Rhinol 2017;7:450-8

4. Brook CD, Maxfield AZ, Ahmed H et al. Factors influencing the need for endoscopic sinus surgery in adult patients with cystic fibrosis. Am J Rhinol Allergy 2017;31:44-7

5. Rosenfeld RM, Piccirillo JF, Chandrasekhar SS, et al. Clinical practice guideline (update): adult sinusitis. Otolaryngol Head Neck Surg 2015;152:S1-S39

6. Halderman AA, Lee S, London NR, et al. Impact of high- versus low-risk genotype on sinonasal radiographic disease in cystic fibrosis. Laryngoscope 2019;129:788-793

7. Tumin D, Hayes D, Kirkby SE et al. Safety of endoscopic sinus surgery in children with cystic fibrosis. Int J Pediatr Otorhinolaryngol 2017;98:25-8

8. Pletcher SD, Goldberg AN, Cope EK. Loss of Microbial Niche Specificity Between the Upper and Lower Airways in Patients with Cystic Fibrosis. Laryngoscope 2019;129:544-50

9. Khalfoun S, Tumin D, Ghossein M, et al. Improved Lung Function after Sinus Surgery in Cystic Fibrosis Patients with Moderate Obstruction. Otolaryngol - Head Neck Surg (United States). 2018;158:381-5

## Physiotherapy

### A28 Effectiveness of energy conservation techniques in the performance of daily life activities in a group of patients with cystic fibrosis

#### Luigi Graziano^1^, Silvia Tucci^2^, Marco Moresi^2^, Marcello Di Paolo^3^, Daniela Savi^3,4^, Vanessa Iuculano^5^, Giada Santoro^2^, Daniela Nico^1^, Tamara Perelli^1^, Giuseppe Cimino^1^, Serenella Bertasi^1^, Donatella Valente^6^, Paolo Palange^3^

##### ^1^Cystic Fibrosis Center, Department of Maternal Infantile and Urological Sciences, Policlinico Umberto I, Sapienza University of Rome, Rome, Italy; ^2^Occupational Therapy BSc Sapienza University of Rome, Rome, Italy; ^3^Department of Public Health and Infectious Diseases, Policlinico Umberto I, Sapienza University of Rome, Rome, Italy; ^4^Cystic Fibrosis Unit, Bambino Gesù Children’s Hospital, Rome, Rome, Italy; ^5^Cystic Fibrosis Center, Department of Adult and Child Human Pathology, University of Messina, Messina, Italy; ^6^Department of Human Neurosciences, Policlinico Umberto I, Sapienza University of Rome, Rome, Italy

###### **Correspondence:** Luigi Graziano (luigi.graziano@uniroma1.it)

**Background**

The aim of pulmonary rehabilitation is to restore the cystic fibrosis (CF) patients to the highest possible level of independence. However, the natural history of CF is characterised by a progressive decline in lung function due to chronic pulmonary infections and recurrent exacerbations [1]. This leads to an increase in symptoms, such as dyspnoea and fatigue, and to intensification of treatments [2]; therefore, the advancement of the illness overshadows the achieving of independence. Energy Conservation Techniques (ECTs), an educational intervention commonly used by occupational therapists, are recommended in the international pulmonary rehabilitation guidelines [3-4] and they help to avoid unnecessary or excessive use of energy during daily life activities (DLA) [5]. The primary objective of this study is to assess the effectiveness of ECTs in terms of reduction of energy expenditure, dyspnoea and fatigue perception.

**Materials and methods**

Seven patients were recruited from April to July 2019 in the Cystic Fibrosis Center (“Policlinico Umberto I” Hospital, Rome). All outcomes were measured during the execution of four tasks, using and not using ECTs: walking, tidying up, dressing, showering. Energy expenditure was measured using the accelerometer SenseWear Pro3 Armband, dyspnea was measured with the modified Borg scale, fatigue was assessed using a ten-point Likert type scale. Oxygen saturation (SpO_2_) and respiratory rate (RR) were also recorded at the end of each task.

**Results**

With ECTs, significant reductions in perceived breathlessness and fatigue scores were reported during walking (1.9±1.5 *vs* 0.8±1.1, p = 0.026; 1.5±1.0 *vs* 0.9±1.1, p=0.034, respectively) and tidying up (1.4±1.0 *vs* 0.5±0.8, p = 0.039; 1.9±0.7 *vs* 0.8±0.7, p<0.001, respectively). Significant decrease in energy expenditure was observed for activities as dressing (32.0±7.6 cal *vs* 22.6±12.5 cal; p=0.041) and showering (40.0±15.0 cal *vs* 32.4±14.5 cal; p=0.015). Walking SpO_2_ also improved with ECTs (93.3±2.7% *vs* 95.4±2.3%; p<0,001) as well as RR during tidying up (20.7±1.7 *vs* 18.1±1.1 breaths/min; p=0.026) , dressing (21.0±1.3 *vs* 18.1±1.6 breaths/min; p=0.027) and showering (20.4±0.8 *vs* 18.3±0.8 breaths/min; p=0.017).

**Conclusions**

Occupational Therapy could supply an innovative contribution in the rehabilitation of patients with CF. It aims to enhance the patient's independence, even in the context of frailty and advanced illness, by adopting Energy Conservation Techniques. Our data suggest that the use of ECTs in CF patients during DLA could reduce energy cost and dyspnoea and muscle fatigue perception, but further research is needed to support the use of energy conservation techniques among CF patients.

**References**

1. Flume PA, Mogayzel PJ, Robinson KA et al. Cystic fibrosis pulmonary guidelines: treatment of pulmonary exacerbations. Am J Respir Crit Care Med 2009;180:802-8

2. Castellani C, Duff AJA, Bell SC, et al. ECFS best practice guidelines: the 2018 revision. J Cyst Fibros 2018;17:153-78

3. Bolton CE, Bevan-Smith EF, Blakey JD, et al. British Thoracic Society guideline on pulmonary rehabilitation in adults. Thorax 2013;68:ii1-30

4. Spruit MA, Singh SJ, Garvey C et al. An official American Thoracic Society/European Respiratory Society statement: key concepts and advances in pulmonary rehabilitation. Am J Respir Crit Care Med 2013;188:e13-64

5. Ip WM, Woo J, Yue SY et al. Evaluation of the effect of energy conservation techniques in the performance of activity of daily living tasks. Clin Rehabil 2006;20:254-61

### A29 Intrapulmonary percussive ventilation (IPV): the effect on the frequency of pulmonary exacerbations in a group of pediatric patients with cystic fibrosis

#### Luigi Graziano^1^, Daniela Nico^1^, Dimitra Kaitsas^2^, Marcello Di Paolo^3^, Daniela Savi^3,4^, Tamara Perelli^1^, Vanessa Iuculano^5^, Sara De Dominicis^2^, Daniele Iorio^2^, Giuseppe Cimino^1^, Serenella Bertasi^1^, Donatella Valente^6^, Paolo Palange^3^

##### ^1^Cystic Fibrosis Center, Department of Maternal Infantile and Urological Sciences, Policlinico Umberto I, Sapienza University of Rome, Rome, Italy; ^2^Physiotherapy BSc Sapienza University of Rome, Rome, Italy; ^3^Department of Public Health and Infectious Diseases, Policlinico Umberto I, Sapienza University of Rome, Rome, Italy; ^4^Cystic Fibrosis Unit, Bambino Gesù Children’s Hospital, Rome, Rome, Italy; ^5^Cystic Fibrosis Center, Department of Adult and Child Human Pathology, University of Messina, Messina, Italy; ^6^Department of Human Neurosciences, Policlinico Umberto I, Sapienza University of Rome, Rome, Italy

###### **Correspondence:** Luigi Graziano (luigi.graziano@uniroma1.it)

**Background**

In patients with cystic fibrosis (CF) the build-up of mucus in the lungs leads to infections and inflammation and eventually to deterioration in lung function [1]. To prevent them, chest physiotherapy is advocated for the clearance of mucus in the airways and the best airway clearance technique should be tailored to the individual [2]. Among these, Intrapulmonary Percussive Ventilation (IPV) is an intrathoracic device that provides continuous oscillation to the airways through the mouth and produces an alternating positive pressure. As a consequence, the vibration loosens the mucus allowing an easier expectoration and therefore improving the airway patency [3]. However, evidence on its efficacy, especially in children population, is still lacking. Therefore, the aim of this study is to assess the efficacy of IPV on the frequency of pulmonary exacerbations (PEx) and the changes in lung function in pediatric patients with CF.

**Materials and methods**

Pediatric CF patients followed at “Policlinico Umberto I” Hospital of Rome and prescribed with IPV treatment between November 2018 to July 2019 were retrospectively recruited. Data regarding number of PEx and lung function (FEV1, FVC, FEF25-75%) were collected and considered as primary and secondary outcomes, respectively. For each patient, comparison between data obtained after starting IPV and those collected from an equal time interval before starting IPV was performed.

**Results**

Nine patients (M/F: 3/6, mean age: 10.2 ± 3.2 years, mean body mass index: 16.5 ± 2.4 kg/m2) were recruited for analysis. Mean length of IPV treatment was 203 ± 94 days (range: 113 – 413 days). Spirometry at the moment of IPV prescription showed a mild-to-moderate impairment in lung function (FEV1: 77.4 ± 8.7% of predicted value). Use of IPV was associated with a significant decline in the rate of PEx (1.7 ± 1.3 with IPV vs 1.0 ± 1.2 before IPV, p = 0.014). A non-significant improvement in mean FEV1 (-1.7 ± 5.0% vs 2.5 ± 3.8% before and during IPV, respectively) and FVC (-1.6 ± 3.7% vs 2.5 ± 3.7% before and during IPV, respectively) changes was observed with IPV.

**Conclusions**

Use of IPV in children with CF is associated with a significant reduction in PEx and a positive trend in lung function. Further prospective studies are needed to confirm these results and to assess the efficacy of IPV on clinical and functional outcomes in CF pediatric populations.

**References**

1. Mall MA. Unplugging Mucus in Cystic Fibrosis and Chronic Obstructive Pulmonary Disease. Ann Am Thorac Soc 2016;13:S177-S185

2. Castellani C, Duff AJA, Bell SC, et al. ECFS Best Practice Guidelines: the 2018 revision. J Cyst Fibros. 2018;17:153-78

3. Lauwers E, Ides K, Van Hoorenbeeck K, et al. The Effect of Intrapulmonary Percussive Ventilation in Pediatric Patients: A Systematic Review. Pediatr Pulmonol. 2018; 53:1463-74

### A30 Physiotherapy in a group of cystic fibrosis patients receiving extracorporeal lung support while awaiting lung transplantation: a retrospective observational study

#### Luigi Graziano^1^, Sabina Martelli^2^, Tamara Perelli^1^, Daniele Diso^3^, Marcello Di Paolo1^4^, Daniela Savi^4,5^, Vanessa Iuculano^6^, Daniele Iorio^7^, Alfredo Storino^7^, Davide Gasparrini^7^, Daniela Nico^1^ Giuseppe Cimino^1^, Serenella Bertasi^1^, Donatella Valente^8^, Paolo Palange^4^

##### ^1^Cystic Fibrosis Center, Department of Maternal Infantile and Urological Sciences, Policlinico Umberto I, Sapienza University of Rome, Rome, Italy; ^2^Department of General Surgery, Surgical Specialities and Organ Transplantation “Paride Stefanini” Policlinico Umberto I, Sapienza University of Rome, Rome, Italy; ^3^Department of Thoracic Surgery, Policlinico Umberto I, Sapienza University of Rome, Rome, Italy; ^4^Department of Public Health and Infectious Diseases, Policlinico Umberto I, Sapienza University of Rome, Rome, Italy; ^5^Cystic Fibrosis Unit, Bambino Gesù Children’s Hospital, Rome, Rome, Italy; ^6^Cystic Fibrosis Center, Department of Adult and Child Human Pathology, University of Messina, Messina, Italy; ^7^Physiotherapy BSc, Sapienza University of Rome, Rome, Italy; ^8^Department of Human Neurosciences, Policlinico Umberto I, Sapienza University of Rome, Rome, Italy

###### **Correspondence:** Luigi Graziano (luigi.graziano@uniroma1.it)

**Background**

Patients with cystic fibrosis (CF) waiting for lung transplantation (Tx) might need advanced therapies as extracorporeal lung support, that can be performed using extracorporeal membrane oxygenation (ECMO) and extracorporeal CO_2_ removal (Prolung®) [1]. These therapies can impede the implementation of a physiotherapy program, so determining a situation of deconditioning that can reduce the chances of survival before and after lung transplantation [2]. Primary aim of this study is to investigate the effect of a physiotherapy program on peri-transplant mortality (i.e. within 60 days) in a group of CF patients that received extracorporeal lung support as a bridge to lung transplantation.

**Materials and methods**

A retrospective observational analysis was conducted on data collected from CF patients admitted to the Transplant Intensive Care Unit of “Policlinico Umberto I” Hospital of Rome from 2010 to 2017, receiving extracorporeal lung support while awaiting lung transplantation. Data were retrospectively collected from medical records: demographic data, tracheostomy status, days of extracorporeal lung support, days in ICU (total and post-Tx) and mortality rates. Patients were divided in two groups according to the level of physical activity (PA) performed during physiotherapy sessions, measured by the ICU Mobility Scale (i.e. low activity ≤ 4 points *vs* high activity > 4 points).

**Results**

Data from twenty patients were obtained for the analysis (13 F; mean age: 30.92 ± 10.32 yrs). Overall mortality was 55%. Among those undergone lung Tx (15/20), peri-transplant mortality was 40% (6/15). Twelve out of twenty patients (60%) performed low levels of PA during their ICU admission. No differences were observed between groups with regards to anthropometric features. Peri-transplant mortality rates differed significantly between groups, being 85.7% *vs* 0% (p = 0.001) among patients with low and high levels of PA, respectively. Furthermore, length of post-transplant ICU stay was significantly higher for patients with low level of PA (27 (22 - 61) *vs* 14.5 (12.2 - 15.7) days, p < 0.001).

**Conclusions**

Higher levels of physical activity may provide better outcomes in terms of peri-transplant mortality and post-transplant ICU stay in CF patients receiving extracorporeal lung support while awaiting lung transplantation. Further studies on a larger sample are needed to fully evaluate benefits and risks of this treatment modality.

**References**

1. King SC, Brown AW, Aryal S et al. Critical Care of the Adult Patient with Cystic Fibrosis. Chest 2019;155:202-14

2. Rehder KJ, Turner DA, Cheifetz IM. Active Rehabilitation During Extracorporeal Membrane Oxygenation as a Bridge to Lung Transplantation. Respir Care 2013;58:1291-8

### A31 Preventive use of nocturnal non invasive ventilation in Cystic Fibrosis: a pilot study

#### Maria Papale, Giuseppe FabioParisi, Sara Manti, Lucia Spicuzza, Enza Mulè, NovellaRotolo, Salvatore Leonardi

##### Respiratory Unit and Cystic Fibrosis, AOU Policlinico-Vittorio Emanuele, Department of Clinical and Experimental Medicine, University of Catania, Via Santa Sofia 78, I-95123 Catania, Italy

###### **Correspondence:** Maria Papale (mariellapap@yahoo.it)

**Background**

In patients with cystic fibrosis (CF) non-invasive ventilation (NIV) improves lung mechanics increasing airflow and gas exchange and decreasing the work of breathing; however, to date, there are no standardized criteria to indicate to whom and when NIV should be started. We investigated whether an early initiation of nocturnal NIV, as a prevention before respiratory failure occurs, affects Lung Clearance Index (LCI) and other clinical and functional outcomes.

**Material and methods**

7 normoxiemic patients (4 males, age 15-34 years, all BMI <18) without history of pneumothorax or presence of blebs were enrolled. All patients were stable at initiation of the treatment. In the first study day spirometry, multiple-breath washout of an inert gas to derive LCI, nocturnal cardiorespiratory polygraphy (PG) were performed. An acclimatization to NIV session, using a bi-level model, to establish the pressures tolerated by each patient was performed.

**Results**

Treatment with NIV significantly reduced nocturnal respiratory rate (*28.4±*4.2 *vs23.5±*1.9) and improved nocturnal SaO_2_ (*91%±*1.0 *vs 94%±*1.0)*,* without affecting nocturnal mean values of TcCO_2_(*38.1±*2.3 *vs 39.1±*2*.*3*,* ns)*.* After one year of nocturnal treatment with NIV, FEV_1_% was stable but the LCI significantly improved (from 17.5 to 15.5). Moreover, the mean number of exacerbation was significantly decreasing during the treatment year (4.7*vs* 2.2, p<0.001). Gas exchange also remained stable as shown by unchanged values of SPO_2_ and TcCO_2_ (Table 1).

**Conclusions**

The early initiation of NIV significantly improved the LCI value, index of global ventilation distribution, and halved the number of exacerbations/year. The novelty of this finding relay in the fact that so far nocturnal NIV in CF has been used during exacerbations or in hypercapnic patients to slow the progression of respiratory failure. A preventive effect of early treatment with NIV has never been suggested. In addition, the effect of NIV on ventilation distribution has never explored before.

**Table 1 (abstract A31).** Demographic and clinical findings of enrolled population at day 1 and after 1 year

Patients	Baseline	During NIV	After 1 year	P value
Age (years)	23.8±6.0			
LCI	*17.4*±3.1		*15.5*±2.7	<0.05
FEV_1_ %	*41.3*±12		*39.9*±2.1	ns
PaO_2_ (mmHg)	*75.5*±12		*73.2*±11	ns
PaCO_2_ (mmHg)	*41.8.*±2.2		*39.0.*±2.1	ns
Awake SpO_2_ (%)	*96*±0.8		*96*±0.4	ns
Awake respiratory rate	*21.7*±2.2		*23.2*±1.8	ns
Exacerbations/year	*4.7*±1.1		*2.2*±0.5	<0.001
**PG Parameters**				
Meannocturnal SpO_2_ (%)	*91*±1.0	*94*±1.0		<0.001
Time with SpO_2_<90%	*9.1*±3.2	*3.4*±1.7		<0.01
Mean TcCO_2_ (mmHg)	*38.1*±2.3	*39.1*±2.3		ns
AHI	*0.6*±0.4	*0.5*±0.4		ns
Mean nocturnal RR	*28.4*±4.2	*23.5*±1.9		<0.01

LCI: Lung Clearence Index; FEV_1_: forced expiratory volume in 1 sec; RR: respiratory rate; PG: poligraphy; TcCO2 : Transcutaneous CO2 ; AHI: apnea-hypopnea index; RR: respiratory rate

### A32 High Flow Nasal Cannula (HFNC): an alternative to Non Invasive Ventilation in Cystic Fibrosis severe lung disease

#### Carlotta Biglia^1^, Alessio Mattei^2^, Roberta Di Tria^1^, Elisa Clivati^1^, Anna Maria Grella^1^, Sara Demichelis^1^, Paolo Maria Trovato^1^, Barbara Messore^1^

##### ^1^Adult Cystic Fibrosis Centre, AOU San Luigi Gonzaga – Orbassano, Torino, Italy; ^2^SCU Pneumologia, AOU Città della Salute e della Scienza di Torino, Torino, Italy

###### **Correspondence:** Carlotta Biglia (carlotta.biglia@gmail.com)

**Background**

Non Invasive Ventilation (NIV) in Cystic Fibrosis (CF) is a bridge to lung transplantation in patients (pts) with end-stage lung disease and a support during acute exacerbations, especially in those with hypercapnia, to avoid ventilator failure. A contraindication for NIV is the occurrence of pneumothorax (PNX), a fearsome complication in advanced CF lung disease. We report our experience using High Flow Nasal Cannula (HFNC) in adult CF pts with respiratory insufficiency secondary to severe lung disease which had to discontinue NIV for spontaneous PNX [1].

**Materials and methods**

3 adult CF patients (1 F, 42 years; 2 M, 28 and 43years) with severe lung disease (FEV1 < 30%, at rest PaO2 50-60 mmHg, PaCO2>45 mmHg), on nocturnal oxygen therapy and NIV (PSV, IPAP 12-15, EPAP 6), chronic respiratory P. aeruginosa infection, > 3 respiratory exacerbations/year, pancreatic insufficiency and BMI < 19; 2 waiting for lung transplantation) presented with spontaneous PNX during an exacerbation (F with a small one < 2 cm, the others 2 with large PNX treated with chest tube)

**Results**

After the onset of PNX patients were switched to HFNC with flow of 25-35L/min, temperature 34-37°C and a FiO2 as required to maintain adequate blood oxygenation (> 93%). Arterial PaCO2 remained stable (<50 mmHg). At time of discharge HFNC was prescribed at home (Fisher and Paykel My Airvo and Optiflow Nasal cannulae). Observational period from acute exacerbation due to PNX ranges from 10 to 28 months: compliance to HFNC is optimal (8 hours/night), blood gases are stable and no concern about safety have raised. Reported comfort was high.

**Conclusions**

HFNC represents a safety alternative to NIV in CF patient with chronic respiratory failure and light compensated hypercapnia, when the use of NIV is not possible as on occurrence of PNX.

**Reference**

1. Sklar MC, Dres M, Rittayamai N et al. High-flow nasal oxygen versus noninvasive ventilation in adult patients with cystic fibrosis: a randomized crossover physiological study. Ann Intensive Care 2018;8:85.

### A33 Occupational therapy: another ally against cystic fibrosis? A survey among patients and carers

#### Luigi Graziano^1^, Giada Santoro^2^, Marcello Di Paolo^3^, Daniela Savi^3,4^, Tamara Perelli^1^, Vanessa Iuculano^5^, Silvia Tucci^2^, Marco Moresi^2^, Daniela Nico^1^, Giuseppe Cimino^1^, Serenella Bertasi^1^, Donatella Valente^6^, Paolo Palange^3^

##### ^1^Cystic Fibrosis Center, Department of Maternal Infantile and Urological Sciences, Policlinico Umberto I, Sapienza University of Rome, Rome, Italy; ^2^Occupational Therapy BSc Sapienza University of Rome, Rome, Italy; ^3^Department of Public Health and Infectious Diseases, Policlinico Umberto I, Sapienza University of Rome, Rome, Italy; ^4^Cystic Fibrosis Unit, Bambino Gesù Children’s Hospital, Rome, Italy; ^5^Cystic Fibrosis Center, Department of Adult and Child Human Pathology, University of Messina, Messina, Italy; ^6^Department of Human Neurosciences, Policlinico Umberto I, Sapienza University of Rome, Rome, Italy

###### **Correspondence:** Luigi Graziano (luigi.graziano@uniroma1.it)

**Background**

Occupational therapy (OT) is a client-centered health profession concerned with promoting health and quality of life through occupation [1]. In the field of respiratory diseases, OT is highly recommended in the care of patients with chronic obstructive pulmonary disease [2], its practice is still not very common in cystic fibrosis (CF). The purpose of this study is to investigate patients’ experience and opinions about OT interventions.

**Materials and methods**

A survey was conducted among patients with CF and their carers using Survey Monkey©. Nine questions have been included, which investigated the level of usefulness of the OT interventions proposed in the survey cover letter. The survey was diffused through the website of the Italian patients’ association (LIFC Onlus), and by sending the link via social network.

**Results**

128 returned surveys were completed by patients (34%) and carers (66%). This survey shows that 83% of patients have never been offered any OT interventions, education in techniques of energy conservation, playful and manual-representative activities for anxiety management or environmental adaptations. The inclusion of OT in the treatment program is considered very helpful by 44% of respondents against the 3% that considered this useless. With regards to the questions on the relevance and usefulness of the other interventions proposed for patients with CF, five options have been included: not at all, not much, enough, greatly, very much. The possibility to benefit from learning and use of energy conservation techniques was considered “enough” by 38% of respondents. The help that the performance of gaming activities and manual-representative activities to alleviate a state of anxiety and the usefulness of devices and environmental adaptations have been considered “greatly” by 37% and 35% respectively of the respondents.

**Conclusions**

The last question of the survey was optional, but despite this there were 102 out of 130 responses. This result shows the great interest that the OT has for CF patients and their carers, as many find it useful for improving the performance of daily life activities and for anxiety management. In particular, the answers show that the OT can be mostly useful to those who have a serious condition or are in an advanced stage of the disease. From these results, we can deduce that there is a great deal of interest by patients to try occupational therapy in CF, for which an implementation in clinical practice is suggested. It is also important to conduct clinical studies on this topic to increase scientific research.

**References**

1. American Occupational Therapy Association. Occupational therapy practice framework: domain & process 3rd edition. Am J Occup Ther 2014;68:S1–S48

2. Bolton CE, Bevan-Smith E, Blakey JD et al. British thoracic society guideline on pulmonary rehabilitation in adults. Thorax 2013;68:ii1

### A34 “F (accio) C (entro)”: project for a smartphone application to increase adherence to aerosol treatment in adolescents with cystic fibrosis

#### Elena Balestri^1^, Sara Vergnani^2^, Stefania Dall’Ara^1^ , Cinzia Grazioli^1^, Benedetta Mannozzi^3^, Maura Ambroni^3^, Fiorella Battistini^3^

##### ^1^U.O.Medicina Riabilitativa, Bufalini Hospital, Cesena, Italy; ^2^University of Bologna, Bologna, Italy; ^3^ Cystic Fibrosis Regional Center, Bufalini Hospital, Cesena, Italy

###### **Correspondence:** Elena Balestri (elenabalestri66@gmail.com)

**Background**

Patients with Cystic fibrosis are daily exposed to a great therapeutic burden represented by respiratory physiotherapy, therapy by aerosol, oral, intravenous and physical activity. Numerous studies underline how during adolescence the therapy adherence, that is undergoing treatments at the right time of the day, in the right sequence and dosage, considerably decreases with negative consequences in terms of: health status, quality of life and hospitalization [1,2]. From these considerations was born “Faccio Centro” the project of a smartphone application for adolescents with CF designed as a tool to increase the adherence to aerosol therapy and physiotherapy guiding them in the process of independence from a parental management of the therapy. Considering the age target, an application has been identified as the most appropriate tool to meet these needs, as the mobile phone is commonly used by adolescents and some studies underline how telemedicine could be a tool to improve the self-management in the FC [3, 4].

**Materials and methods**

“**F**accio **C**entro” is a project who integrating the results of the research conducted on the main biomedical databases, google scholar, apple store, android store and the interview with adolescents patient of the “cystic fibrosis centre” of the Bufalini Hospital in Cesena. In the app’s homepage the daily therapies appear in the temporal order of execution with notes about the correct assumption modalities, a graph indicates the percentage of therapy carried out and there is written the date of the next check with a customizable reminder attached. The patients can set a reminder alarm for each therapy and check if they have performed it or not and the checks will no longer be modifiable after midnight of the same day. In other sections user can interact autonomously with the professionals of the centre by sending an email directly to the most suitable figure to answer his question (doctor, nurse, physiotherapist) and also they can download a summary table of therapies conducted each month with indications on the trend of adherence.

**Conclusions**

The “**F**accio **C**entro” APP designed to be tailored to the patients, simple, customizable, engaging and interactive could be a support tool for adolescents in the autonomous management of therapy and to contain the decline in adherence that has been recorded in the transition from childhood to adolescence. The next step will be to create a prototype of the APP for the patients of the centre to assess its impact on adherence.

**References**

1. Quittner AL, Zhang J, Marynchenko M, et al. Pulmonary Medication Adherence and Health-care Use in Cystic Fibrosis. Chest 2014;146:142-51

2. Eakin MN, Riekert KA. The impact of medication adherence on lung health outcomes in cystic fibrosis. Curr Opin Pulm Med 2013;19:687–91.

3. Ketchell RI. Telemedicine is the way forward for the management of cystic fibrosis – the case in favour. Paediatr Respir Rev 2018;261:9–21.

4. Lomas P. Enhancing adherence to inhaled therapies in cystic fibrosis. Ther Adv Respir Dis 2014;8:39–47

## Quality improvement

### A35 External Quality Assessment in Cystic Fibrosis Genetic Testing: the Italian Experience

#### Federica Censi^1^, Fabrizio Tosto^1^, Marco Salvatore^1^, Ave Maria Baffico^2^, Alessandra Coiana^3^, Marco Lucarelli^4^, Elisabetta Pelo^5^, Manuela Seia^6^, Domenica Taruscio^1^

##### ^1^Centro Nazionale Malattie rare Istituto Superiore di Sanità, Roma, Italy; ^2^IRCCS Istituto Giannina Gaslini, U.O.C. Laboratorio di Genetica Umana, Genova, Italy; ^3^Dipartimento di Scienze Mediche e Sanità Pubblica, Università di Cagliari; Laboratorio di Genetica e Genomica, Azienda Ospedaliera Brotzu; Ospedale Pediatrico Microcitemico “A. Cao”, Cagliari, Italy; ^4^Dipartimento di Biotecnologie Cellulari ed Ematologia” in “Dipartimento di Medicina Sperimentale, Sapienza Università di Roma– Azienda Policlinico Umberto I, Roma, Italy; ^5^SOD Diagnostica Genetica, AOU Careggi, Firenze, Italy; ^6^Laboratorio di Genetica Medica, Fondazione IRCCS Policlinico Cà Granda Ospedale, Milano, Italy

###### **Correspondence:** Federica Censi (federica.censi01@iss.it)

**Background**

Molecular analysis of CFTR gene is a key step in the diagnosis of Cystic Fibrosis (CF), carrier testing and prenatal diagnosis; it represent the most frequent genetic test carried out in Italy [1]. An error in the genotype analysis or an incorrect or inaccurate interpretation may have consequences on the life choices of patients and their family. In this context, it is essential that laboratories are competent to perform tests at an acceptable standard and to make sense of the information provided by the test. Monitoring genetic laboratories is an obligation for the National Health System as part of its mandate to protect the health and quality of life of citizens. The Italian External Quality Assessment (IEQA) for Cystic Fibrosis started in 2001; it was coordinated by the Istituto Superiore di Sanità (ISS) [2;3]. The aim of the activity is to monitor and to improve quality of genetic testing performed by Italian laboratories. In 2009 the activity was published in the Official Bulletin of the Italian Republic [G.U. n.199 del 28/08/2009] and a marking system was established.

**Materials and methods**

Participation is voluntary and open to both public and private Italian laboratories. Laboratories pay a fee to participate. The IEQA scheme organizer and national experts provide advice on the scientific context of the scheme and take decisions and educational actions for the development of the programme.ISS provides four validated samples of genomic purified DNA, annually. All samples have been selected to represent typical mutations of the gene; they are distributed with mock data identifications, mock clinical patient detail and technical data. Poor performance was marked since 2013.All data are managed through a web utility designed to simplify communication and data sharing among ISS, laboratories and assessors.

Laboratories are asked to process samples and to return results of genotyping and a full interpretative report. Scheme is strictly anonymous. Five National assessors evaluate laboratory results, according to established criteria

**Results**

Until now 15 rounds have been completed and overall 90Italian laboratories have been monitored. Participants laboratories returned acceptable analytical results, in recent years compared to previous ones, but we still registered a number of reports with not complete or not accurate interpretation.

**Conclusions**

Our observation highlight that laboratories that constantly participate to the EQA return more complete and accurate results. EQA should be viewed as educational tool and used to help direct improvement efforts in the laboratory

**References**

1. Giardino D, Mingarelli R, Lauretti T et al. Survey of medicalgeneticservices in Italy: year 2011. BMC Health Serv Res 2016;16:96

2. Taruscio D, Falbo V, Floridia G et al. Quality assessment in cytogenetic and molecular genetic testing: the experience of the Italian Project on Standardisation and Quality Assurance. Clin Chem Lab Med 2004;42:915-21

3. Censi F, Tosto F, Floridia G et al. The Italian National External quality assessment program in molecular genetic testing: results of the VII round (2010-2011). Biomed Res Int. 2013;2013:739010

### A36 Single versus bilateral sweat chloride testing

#### Natalia Cirilli, Benedetta Fabrizzi, Nicole Caporelli, Anastasia D’Antuono, Tiziana Brasili, Nadia Baiocco, Marco Cipolli

##### Cystic Fibrosis Centre, Mother-Child Department, United Hospitals, Ancona, Italy

###### **Correspondence:** Natalia Cirilli (natalia.cirilli@ospedaliriuniti.marche.it)

**Background**

The diagnosis of cystic fibrosis (CF) requires confirmatory diagnostic tests that should always include a sweat test, even when two CF-causing mutations are identified. Sweat testing requires experienced staff who should follow standard operating procedures. There are clear guidelines available for laboratories providing a sweat test service that recommend to collect sweat from both arms to decrease the probability of having an insufficient sweat quantity. Some evidences were published regarding the real impact of this practice. In our CF centre we changed our procedure in January 2018 from sweat test on one arm to sweat test on both arms. To evaluate the effectiveness of these two procedures we compared data.

**Materials and methods**

In our centre we regularly perform the sweat test using the Macroduct modified method (Iontophoresis stimulation with pilogel discs, collection on filter paper, chloride titration by coulometry). We compared sweat chloride records in 2 different periods: June 2016 – December 2017 (single sweat test) and January 2018 – July 2019 (bilateral testing). In these two periods the technical staff dedicated to sweat testing was the same. Our CF centre perform internal quality control as recommended (mean CV% for all 3 levels: 6.85%) and participate in the national External Quality Assessment scheme (overall performance in 2018: 100%). Statistical analyses included Chi-square test at 0.05 level of significance.

**Results**

Results are showed in Table 1

These results show that collecting sweat from both arms don’t improve the proportion of tests with sufficient sweat weight; anyway bilateral testing could be used as internal quality control.

**Conclusions**

Sweat testing is a crucial laboratory test for CF diagnosis. To maintain a high quality performance of this test it’s mandatory to retrain the technical staff and check the equipment regularly. A single test procedure can guarantee a good sweat test performance taking also into account the reimbursement of the test which is far from the real cost.

**Table 1 (abstract A36).** Comparative analysis

	Single sweat test		Bilateral sweat test	
subjects with QS	310	92.8%	229	92.0%
subjects with at least 1 QNS	19	5.7%	14	5.6%
subjects with 2 or more QNS	5	1.5%	6	2.4%
subjects tested	334	100.0%	249	100.0%

QS=sweat quantity sufficient; QNS=sweat quantity not sufficient

Chi square test = 0.642; p=0.725

## Psychology

### A37 A proposal for a new assessment tool of burnout in italian CF healthcare workers

#### Silvia Bresci^1^, Claudia Giust^2^, Giuseppe Scopelliti^3^, Riccardo Guarise^4^, Rosaria Casciaro^5^, Andrea Gramegna^6^, Paola Iacotucci^7^, Barbara Messore^8^, Giovanna Pizzamiglio^6^, Elena Spinelli^4^

##### ^1^SOD Malattie Infettive e Tropicali, AOU Careggi, Firenze, Italy; ^2^ SOS Psychology, Ospedali Riuniti, Ancona, Italy; ^3^Cystic Fibrosis Referral Care Center, Mother-Child Department, Ospedali Riuniti, Ancona, Italy; ^4^Cystic Fibrosis Regional Centre, Azienda Ospedaliera Universitaria Integrata Verona, Italy; ^5^CysticFibrosisReferral Care Center, IRCCS Giannina Gaslini, Genova, Italy; ^6^University of Milan, Department of Pathophysiology and Transplantation; Fondazione IRCCS Ca’ Granda Ospedale Maggiore Policlinico, Internal Medicine Department, Respiratory Unit and Cystic Fibrosis Adult Center, Milano, Italy; ^7^Cardiovascular Emergency Department, Clinical Medicine and Aging Medicine, Cystic Fibrosis Referral Adult Care Centre, Federico II, Napoli, Italy; ^8^Cystic Fibrosis Referral Adult Care Centre, AOU S Luigi Gonzaga, Orbassano-Torino, Italy

###### **Correspondence:** Silvia Bresci (brescis@aou-careggi.toscana.it)

**Background**

The assessment of burnout is important to understand the wellness of healthcare workers and the quality of their performance. There are many tests for the analysis of burnout, such as the Maslach Burnout Inventory (MBI) [1], which are usually generic and not specific for workers that deal with chronic diseases, in particular with Cystic Fibrosis (CF). The aim of this project is to implement MBI tool with CF-related items to better understand burnout phenomenon in healthcare workers that take care of patients with Cystic Fibrosis and point out its characteristics.

**Materials and methods**

After a literature review [2,3] and analysis, specific fields were selected by psychologists focus group and proposed to multiprofessional group of Adult Committee of Italian Cystic Fibrosis Society.

**Results**

New nine statements were chosen, divided by three main psychological items (Powerlessness, Framework, Contention) and they will be presented to CF community of healthcare professionals, in addition to the M.B.I. A subgroup analysis will be performed to validate clinimetric properties of the new version of CF-related MBI. Demographic and job related data were included.

**Conclusions**

The introduction of a questionnaire which is specific for the pathology could be a more accurate evaluation tool to assess burnout in CF healthcare workers, with the aim of finding and treating in advance the discomfort of the individual worker, but also of being able to efficiently organize the team. Therefore, our purpose is to present this test to Italian CF healthcare workers to understand its effectiveness and its benefits in clinical practice.

**References**

1. Sirigatti S.&Stefanile, C. Adattamento e taratura per l’Italia. In C. Maslach & S. Jackson, MBI Maslach Burnout Inventory. Manuale. Firenze Organizzazioni Speciali. 1993:33-42

2. Lewiston NJ, Conley J, Blessing-Moore J. Measurement of hypothetical burnout in cystic fibrosis caregivers. Acta Paediatr Scand. 1981;70:935-9

3. Goldbeck L, Fidika A, Heuer HE, et al. Professional quality of life among CF healthcare providers. J Cyst Fibros 2015;14:S17

## Comorbidities

### A38 Early detection of glucose derangements in children with cystic fibrosis

#### Giuseppina Napoletano^1^, Emanuela Rossitti^1^, Anna Coruzzo^1^, Angela Sepe^1^, Antonella Tosco^1^ Enza Mozzillo^2^, Valentina Fattorusso^3^, Adriana Franzese^3^, Valeria Raia^1^

##### ^1^Cystic Fibrosis Center, Department of Translational Medical Sciences University of Naples Federico II, Naples, Italy; ^2^Section of Pediatrics, Regional Center of Pediatric Diabetology, Clinical Department of Maternal and Child Health, University of Naples Federico II , Naples, Italy; ^3^Section of Pediatrics, Regional Center of Pediatric Diabetology, Department of Translational Medical Science, University of Naples Federico II Naples, Italy

###### **Correspondence:** Giuseppina Napoletano (dr.giusinapoletano@gmail.com)

**Background**

Cystic Fibrosis Related Diabetes (CFRD) adversely affects pulmonary function, nutrition and survival. It’s prevalence ranges from 9% at age <10 years to 35-40% at age >20 years. According to standard of care, annual screening for CFRD with OGTT should begin by age 10 years. Recently a prevalence up to 40% of altered glucose tolerance between 6 and 10 years, predictive of early evolution in CFRD, has been detected. The present study aims to find and monitor early glucose derangements in CF patients.

**Materials and methods**

We retrospectively collected data regarding children with CF in stable clinical condition annually evaluated by OGTT. Patients were classified according to ISPAD 2018 criteria: CFRD, fasting glucose > 126 mg/dl; CFRD, Non Fasting Hyperglicemia (CFRD-nonFH), 2 hours postload glucose > 200 mg/dl during OGTT; Impaired Glucose Tolerance (IGT), 2 hours postload glucose 140 to 199 mg/dl during OGTT; Indeterminate (INDET), fasting glucose < 126 mg/dL and 2 hours postload glucose < 200 mg/dL, but with glucose > a 200 mg/dl during intermediate times at OGTT (T30-T90 minutes). Moreover we classified as Abnormal Glucose Tolerance 140 (AGT140) those subjects with glucose 140 to 200 mg/dl at intermediate times of OGTT.

**Results**

Data from 72 patients (51,4% F), 318 total OGTT, were collected. Mean age was 6.2±1.96 ys at first OGTT (range 3.7-9.5 ys). At the time of the first observation 50/72 (69.4%) showed glucose derangements: 3 CFRD (4,2%), 16 IGT (22.2%), 5 INDET (6.9%), 26 AGT140 (36.1%). 6/72 started insulin therapy (3 CFRD, 3 IGT). 59/66 (89.3%) subjects who did not start insulin therapy, annually performed an OGTT for a mean follow up of 5.8±3.4ys. At the last OGTT, 42/59 (71.2%) showed a glucose derangement: 6/59 (10.2%) CFRD (mean age: 11±2,3 yrs), 11 IGT (18.6%), 5 INDET (8.5%), 20 AGT140 (33.9%). During follow-up no patient with normal glucose tolerance at first OGTT developed CFRD, while 9/24 AGT140 group (37.5%) developed IGT/CFRD. Preliminary data showed that there was no progression towards glyco-metabolic cathegories in patients who performed insulin therapy.

**Conclusions**

The high prevalence of glyco-metabolic derangements in CF children < 10 years of age suggests the relevance of OGTT as a metabolic screening tool before 10 years, differently from what reported in current recommendations. Moreover, the potential evolution to CFRD also for AGT140 group, not yet identified as at risk for glyco-metabolic derangements, suggests the need of longer-term studies to better define evolution of different glucose alterations in CF children.

### A39 Prevalence of urinary incontinence in female with cystic fibrosis followed at the Cesena’s CF Regional Centre

#### Elena Balestri^1^, Virginia Magnani^2^, Stefania Dall’Ara^1^, Cinzia Grazioli^1^ , Maura Ambroni^3^, Fiorella Battistini^3^

##### ^1^U.O. Medicina Riabilitativa, Bufalini Hospital, Cesena, Italy; ^2^University of Bologna, Bologna, Italy; ^3^Cystic Fibrosis Regional Center, Bufalini Hospital, Cesena, Italy

###### **Correspondence:** Elena Balestri (elenabalestri66@gmail.com)

**Background**

Urinary incontinence is today a recognized problem and it is reported in various studies in literature^1^. The main cause of UI is chronic cough, which leads to a progressive weakness of the pelvic floor muscles. The aim of the study is to evaluate the prevalence of urinary incontinence in patients with cystic fibrosis followed at the Cesena’s CF Regional Centre and to determine the presence or absence of a correlation between incontinence and age, body mass index and lung function.

**Materials and methods**

The International Consultation on Incontinence Questionnaire Short Form^2^ (ICIQ-UI SF) was administered to female patients aged 10 years and older during routine visits, in a period from November 2018 to May 2019. Patients who received lung transplantation were excluded from the study. Clinical data of participants were collected including height, weight, forced vital capacity (FVC), forced expiratory volume in one second (FEV1) and expiratory flow medium forced (MEF 75/25). FVC, FEV1 and MEF 75/25 were measured using spirometry, performed on the same day the questionnaire was administered.

A statistical analysis was carried out by t-test, a significance level of 95% was considered.

**Results**

Forty-nine (98%) of 50 eligible patients (age: 10–53 years) participated. Twenty patients (41%) reported urinary incontinence. The presence of urinary incontinence was associated with increasing age. There is a correlation between incontinence and lung function (measured by FEV1 and MEF 75/25), although it’s not statistically significant (p = 0.06). No correlation was found between UI and body mass index or lung function measured by FVC (Table 1). All incontinent female reported stress UI; the situations that most commonly determine urine losses are coughing/sneezing, physical activity and laughing respectively in 80%, 20% and 10% of the patients. 94% of patients reported that the incontinence doesn’t impact on their daily life.

**Conclusions**

Urinary incontinence is a frequent and underestimated condition that commonly affects women with cystic fibrosis, although this problem is often not reported by patients to their physician or physiotherapist, probably due to embarrassment. It’s important to identify patients with incontinence because simple exercises to strengthen the pelvic floor muscles can improve the situation^3^. Investigating the presence of UI should become part of routine visits.

**References**

1. Frayman KB, Kazmerski TM, Sawyer SM. A systematic review of the prevalence and impact of urinary incontinence in cystic fibrosis. Respirology 2018;23:46-54

2. Tubaro A, Zattoni F, Prezioso D et al. Italian validation of the International Consultation on Incontinence Questionnaires. BJU Int. 2006;97:101-8

3. Qaseem A, Dallas P, Forciea MA et al. Nonsurgical Management of Urinary Incontinence in Women: A Clinical Practice Guideline From the American College of Physicians. Ann Intern Med. 2014;161:429.

**Table 1 (abstract A39).** Clinical data of the Study Population

Characteristics	Incontinent (n=20)	Continent (n=29)	p-value
Age (years)	27.4 (13.98)	18.14 (7.52)	*0.01*
BMI (kg/m2)	20.41 (4.03)	20.01 (3.03)	0.70
FEV1 (% predicted)	74.29 (26.65)	86.91 (27.03)	0.06
FVC (% predicted)	87.39 (20.55)	95.10 (20.20)	0.20
MEF75/25 (% predicted)	48.06 (33.56)	63.83 (37.03)	0.06

Data are shown as mean with standard deviation

## Case reports

### A40 A rare association: Cystic fibrosis in patient with Down syndrome

#### Francesca Ficili, Annalisa Ferlisi, Lisa Termini, Maria Antonietta Orlando, Gabriella Traverso, Sabrina La Fata, Caterina Lo Piparo, Mirella Collura

##### Cystic Fibrosis Center and Chronic Respiratory Diseases, O.U. Second Pediatrics “Di Cristina Hospital”, ARNAS CIVICO, Palermo, Italy

###### **Correspondence:** Francesca Ficili (fficili@hotmail.com)

**Background**

Cystic fibrosis (CF) is a multisystemic hereditary, incurable and chronic disease which causes severe damages to respiratory and digestive tracts. It is the most common genetically inherited disease among caucasians. This disease is caused by defects in CF genes, the so-called mutations in cystic fibrosis transmembrane conductance regulator (CFTR) gene population.

**Case Report**

R.D.G. is a SGA newborn with signs of fetal malnutrition, clinical/dysmorphisms features compatible with Down Syndrome, born at 38.1 weeks of gestational age with caesarean section. Birth weight 2.035 kg. At birth he showed a mild respiratory distress and difficulty in feeding so was admitted to NICU for a month. Because of the presence of dysphormic features it has been performed a standard karyotype that showed 47 chromosomes and the presence of trisomy 21. The patient was asked to repeat a neonatal screening for abnormal value of IRT and a sweat test, which wasn’t performed because of recurrent bronchitis. After the discharge the patient showed many episodes of respiratory infections including some episodes of bronchitis with fever and cough. At the age of seven months, he was admitted to Hospital for cough, fever and dyspnea; it was therefore started empirical antibiotic therapy, first with amoxicillin/clavulanic acid then replaced with clarithromycin, but with very slow improvement. For an improved clinical evaluation, the patient had undergone further assessment: there were performed sweat tests that led to pathological results: 128 mEq/L, 137 mEq/L and 138 mEq/L. Chymotripsine and fecal elastases were abnormal in multiple evaluations. Chest X-ray showed multiple pneumoniae. Coltural sputum showed the presence of S. Maltophilia so we started intravenous cefalosporine with clinical improvement. Genetic investigations confirmed the presence of mutations compatible with cystic fibrosis: F(508)del/ F(508)del. The family has been trained in respiratory physiotherapy and the patient started pancreatic enzymes with improved growth and respiratory symptoms.

**Conclusion**

The combination of two genetic pathologies with an unfavorable prognosis is very uncommon. In literature the association of cystic fibrosis and down syndrome is rare and accidental.

Parents gave consent to patient data publication.

### A41 A complicated association between two different genetic rare disorders: Spinal Muscular Atrophy and Cystic Fibrosis

#### Paolo Buonpensiero^1^, Marta Palma^1^, Simona Spadarella^1^, Bernadette Donnarumma^1^, Giada Zollo^1^, Francesco Nunziata^1^, Chiara Cimbalo^1^, Alice Castaldo^2^, Gaetano Terrone^3^, Antonio Varone^4^, Antonella Tosco^5^, Angela Sepe^5^, Valeria Raia^5^

##### ^1^Department of Translational Medical Science, Section of Pediatrics, University Federico II, Naples, Italy; ^2^Department of Public Health, University of Naples “Federico II”, Naples, Italy; ^3^Department of Medical and Translational Sciences, Child Neuropsychiatry, Federico II University, Naples, Italy; ^4^Department of Neuro-sciences, “Santobono-Pausilipon” Hospital, Naples, Italy; ^5^Regional Cystic Fibrosis Center, Pediatric Unit, Department of Translational Medical Sciences, Federico II University, Naples, Italy

###### **Correspondence:** Paolo Buonpensiero (pbuonpensiero@gmail.com)

**Background**

Type 1 Spinal Muscolar Atrophy (SMA1) is a genetic disorder that affects the spinal motor neuron; the most common form is an autosomic recessive defect of the survival motor neuron gene 1 (SMN1); it generally onsets before 6 months of life presenting with severe hypotonic weakness in the lower limbs, respiratory distress, weak cry, and poor feeding [1]. In several cases Cystic Fibrosis (CF) poor nutritional status is associated to severe malabsorption. In this case the neuromuscular manifestations may involve legs with numbness, tingling, pain, weakness and unsteadiness of gait. We describe a rare case of a patient affected both by CF and SMA1 associated to concomitant clinical manifestations .

**Case Report**

A female 13 months-old was affected by CF with pancreatic insufficiency, diagnosed through a positive NBS and two CF-causing mutations [F508del/4016insT] at CFTR genetic analysis. In the first months of life, she was hospitalized for recurrent respiratory infections, poorly responsive to conventional treatment with oral/intravenous antibiotics and physiotherapy. Contemporary the patient showed a poor growth status despite PERT and progressively a severe hypotonia with delayed acquisition of developmental milestones, not elicitable osteotendinous reflexes, tongue fasciculations with lack of cough reflex. Based on these symptoms Motorplex panel (analysis of genes causing muscle disorders) was performed resulting in a homozygous deletion for SMN1, compatible with diagnosis of type 1 SMA. A modified personalized physiotherapy program was promptly started, including airway clearance techniques with intrapulmonary percussive ventilation (IPV) and mechanical insufflation-exsufflation. Total enteral feeding by percutaneous endoscopic gastrostomy (PEG) was set up with improvement of clinical and nutritional conditions. The patient started experimental therapy with Nusinersen, a modified antisense oligonucleotide that increases the production of full-length SMN protein, approved for intrathecal use in paediatric and adult patients with SMA. It has been demonstrated that early treatment of this drug is crucial [2] to improve motor development in SMA.

**Conclusion**

Based on our knowledge, this is the first case in which these two genetic diseases occur in the same patient. The progressive neuromuscular weakness that characterizes SMA may impact on delayed mucociliary clearance affecting progressive lung disease and frequent pulmonary exacerbations. IPV is an adequate alternative to conventional chest physiotherapy in this case, also in order to impact upper airway muscle weakness and spinal deformity. We hope that therapy with Nusinersen, and a continuous personalized physiotherapy program may impact on natural history of both diseases and potentially on survival. Patient’s parents gave consent for the publication of clinical data.

**References**

1. Arnold WD, Kassar D, Kissel JT. Spinal muscular atrophy: diagnosis and management in a new therapeutic era. Muscle Nerve 2015; 51:157-67

2. Hoy SM. Nusinersen: First Global Approval. Drugs 2017; 77:473-9

### A42 An unusual case of pulmonary atelectasis and cytomegalovirus infection

#### Piercarlo Poli^1^, Silviana Timpano^1^, Francesca Caldarale^2^, Luisa Giannone^2^, Rita Padoan^1^, Raffaele Badolato^3^

##### ^1^Regional Support Center for Cystic Fibrosis, Department of Pediatric, ASST-Spedali Civili of Brescia, Brescia, Italy; ^2^Department of Pediatric, University of Brescia, Brescia, Italy; ^3^Department of Pediatric, University of Brescia, ASST-Spedali Civili of Brescia, Brescia, Italy

###### **Correspondence:** Piercarlo Poli (piercarlo.poli@gmail.com)

**Background**

N.T. is a 6-month-old infant, born at full term with a normal perinatality. At 15 days of life he was referred to our Center for positive neonatal screening for Cystic Fibrosis (CF) with two F508del CFTR mutations. The positive result of the sweat test (Chloride 64 mEq/L) confirmed the diagnosis and at the age of 17 days he was taken in charge by our CF Center of Brescia. Fecal pancreatic elastase has also documented exocrine pancreatic insufficiency.

**Case Report**

At the age of four months, for persistent cough and polypnea, he performed a chest X-ray with an atelectasis of the right upper lobe and bilateral interstitial infiltrates. N.T. was then admitted to our Department for intravenous antibiotic therapy with Cefotaxime (last naso-pharyngeal aspirate culture: methicillin sensitive Staphylococcus aureus, present in respiratory secretions already from the first weeks of life). On the second day of hospitalization, due to the worsening of respiratory symptoms, we supported ventilation with high flow nasal cannula; but for the persistence of the atelectasis on chest X-ray, it was decided to have a bronchoscopy with bronchoalveolar lavage (BAL) with Dornase alfa (Pulmozyme). During the endoscopic session tenacious dense mucous secretions were aspirated, especially from the right bronchus. Chest X-ray after 72 hours from bronchoscopy showed a clear improvement in the ventilation of the right upper lobe with an almost complete resolution of the atelectasis. The bacteriological and virological tests performed on the BAL were positive for high-load cytomegalovirus (CMV) (CMV DNA: 10.228.432 copies/ml) as per current lung infection. Therefore intravenous therapy with Ganciclovir (5 mg/kg/day) was started for 15 days. Plasma and nasopharyngeal aspirate CMV copies were also high (15.021 copies/ml, 243.703 copies/ml, respectively). Congenital CMV infection was excluded through the negative CMV DNA on the Guthrie card conserved at the Regional Neonatal Screening Laboratory (Buzzi Hospital, Milan) and through the serology that confirmed the presence of specific IgM. The child was discharged after 25 days of hospitalization with negative CMV plasma copies. Subsequent respiratory culture tests were negative for CMV.

**Conclusion**

In our experience the improvement of the radiological picture is to be attributed to Pulmozyme during bronchoscopy. For CMV pulmonary infections in immunocompetent CF patients it is advisable to include the search for this infection in routine diagnostic practice and it should be indicated to dose CMV DNA from the nose-pharyngeal aspirate/sputum/serum in cases of pulmonary exacerbation not responding to conventional therapies and start, if positive, specific antiviral therapy^1-2^. Patient’s parents gave consent for the publication of clinical data.

**References**

1. Parkins MD, Ramos KJ, Goss CH, et al. Cytomegalovirus: an unrecognized potential contributor to cystic fibrosis disease progression? Eur Respir J 2019;53. doi: 10.1183 / 13993003.01727-2018

2. Sawant A, Spoletini G, Whitaker P, et al. Cytomegalovirus-associated pulmonary exacerbation in patients with cystic fibrosis. ERJ Open Res 2018;4. doi: 10.1183 / 23120541.00111-2017

## Nursing

### A43 Evaluation of “home nursing service” for intravenous antibiotics administration in cystic fibrosis patients: 1-year experience in Livorno

#### Francesca Nistri^1^, Matteo Botti^2^, Amalia Negri^3^, Sabrina Quinti^3^, Lucia Gadducci^1^, Ivan Querci^1^, Paola Giostra^3^, Giuseppina Pisano^3^, Paola Catastini^3^, Roberto Danieli^3^

##### ^1^Nurses of “home nursing service”, financed by monetary Fund of Tuscany Region dedicated to Cystic Fibrosis, SVS-Pubblica Assistenza, Livorno, Italy; ^2^Department of Pediatric, University of Pisa, Santa Chiara Hospital, Pisa, Italy; ^3^Regional Support Center for Cystic Fibrosis, Department of Pediatric, Livorno Hospital, ASL Nord-Ovest Toscana, Livorno, Italy

###### **Correspondence:** Francesca Nistri (francesca.nistri@hotmail.it)

**Background**

Endobronchial infections in cystic fibrosis (CF) can require treatment with intravenous (i.v.) antibiotics for several days in the hospital, affecting health costs and quality of life for patients and their families. Home i.v. therapy can be an equally effective alternative^1^; in Italy usually the patient has to manage, prepare and administer the therapy himself or with the help of caregiver. Home care of qualified nurses (“*home nursing service*”, HNS) can lead to an improvement of assistance, quality of life and therapeutic compliance^2^.

**Materials and methods**

In Livorno, since August 2018, patients who needed i.v. antibiotic were offered the HNS, seven days a week, to reconstitute and administrate i.v. drugs. Three qualified nurses trained in CF, performed the HNS. In every patient, we evaluated, with a written anonymous questionnaire the acceptance and satisfaction of HNS, the compliance with the prescribed therapeutic duration, the number and type of adverse drug reactions and the procedural anxiety.

**Results**

We enrolled 6 adult with CF (median age: 26,5 years), in follow-up in Livorno CF Support Centre, to receive HNS. In previous years, all these had already received i.v. therapy at home, with the help of a caregiver (usually a trained family member). In this year 10 antibiotic cycles were performed overall with HNS; during i.v. therapy 5/6 patients had peripheral venous catheter (PVC), 1/6 had central venous catheter. Five patients immediately accepted the HNS gladly, while one accepted afterward, for the initial fear of “privacy violation”. Contrary to the past, the therapeutic compliance was complete: always the prescribed duration of i.v. therapy has been maintained. None had any allergic drugs reactions; in two cases, with PVC, the presence of the nurse at home has allowed detection of early signs of phlebitis. HNS satisfaction was assessed positively by all patients (median score: 4,8/5). The level of procedural anxiety before the introduction of HNS and during HNS (self-declared with the questionnaire), showed a reduction (median scores: 3,8/5 before HNS and 1,2/5 during HNS).

**Conclusions**

The results of this preliminary study showed that all patients are satisfied with their current HNS. The nurses played an important role in improving the home i.v. therapy by supervising the patient and identifying precociously the potential problems. Our experience, which we have intention to expand with other evaluations, suggests that HNS provides a positive link between the hospital and patient’s home life, reduced the anxiety and improves the therapeutic compliance.

**References**

1. Balaguer A, González de Dios J. Home versus hospital intravenous antibiotic therapy for cystic fibrosis. Cochrane Database Syst Rev. 2015;12:CD001917

2. Elsey L. Service evaluation of a cystic fibrosis home intravenous antibiotic service provided by a NHS foundation trust. Arch Dis Child. 2016;101:e2

### A44 Cystic fibrosis patient: proactive approach for venous heritage preservation

#### Chiara Ronca^1^, Ornella Panico^2^, Amelia Cherubin^1^, Ersilia Licciardi1, Elisa Marconi^1^, Andrea Palazzin^1^, Silvia Antoniazzi^1^, Roberta Beghini^1^, Rosanna Demas^1^, Marta Marchetti^1^, Ornella Rigo^1^, Carla Seno^1^, Eva Tinazzi^1^, Nicoletta Trevisani^1^, Aurora Viglio^1^, Giacoma Scorca^1^, Giovanna Amenta^1^, Silvia Mancuso^1^, Daniela Venturini^1^, Sergio De Nardi^3^

##### ^1^Cystic Fibrosis Centre, Azienda Ospedaliera Universitaria Integrata, Verona, Italy; ^2^Università degli studi di Verona, Verona, Italy; ^3^BARD/BD Milano, Italy

###### **Correspondence:** Chiara Ronca (chiara.ronca@aovr.veneto.it)

**Background**

Cystic Fibrosis (CF) patients are admitted to hospital during their lifetime. In hospital, and at home, the drugad ministration frequently takes place intravenously. According to a proactive approach, a single vascular device should be placed, thus allowing to complete the whole diagnostic-treatment pathway. The benefits of such a choice have consequences both on the quality of life of the patient and on cost saving: nurses won’t waste time looking for a new vascular access, and there will be a reduction in phlebitis, infections and extravasations that can occur when numerous attempts to cannulate are necessary. Patients with chronic diseases are usually mistrustful towards changes, in particular about therapies or devices. In consideration of the fact the CF patient should benefit from a central access, due to the kind of drugs to administer, a compromise had to be reached. The nurses, sometimes have more difficulty in accepting new procedures or devices for their patients or for the work organization. A new vascular device needs a time of placement which cannot be very short. The aim of our work is to reduce the number of venipunctures and improve the quality of life of patients.

**Materials and methods**

- A long cannula has been chosen, power-injectable and in soft polyurethane so that it could be placed both in superficial and deep veins and could be used for blood collection too.
- Evaluation of the problem has been detected.
- National and international guidelines have been consulted.
- Getting in touch with devices producers has been made, as well as choice of the device (power glide).
- Theoretical and practical training have been carried out by the Clinical Specialist of the producer (BARD).
- Tutorship with Clinical Specialist until autonomy has been followed.
- Purchase of Ultrasound for venipuncture guidance has been used.

**Results**

Patients ‘point of view: Satisfaction for the dwell time of the device; they report less pain during placement; new patients asked for the device when admitted to hospital. Nurse ‘point of view: satisfaction for the dwell time of the device; decrease in the number of venipunctures; increase in technical skills (US guidance during venipuncture).

Preliminary data from 140 observations have the following results: average dwell time: 16 days; reasons for removal; end of therapy: 51%; thrombophlebitis: 4.4%; hematoma: 0.6%; pain: 2.5%; displacement: 2.5%; pre-dismissal change: 0.6%; malfunction: 3.1%.

**Conclusions**

Following suggestions:

- complications survey: phlebitis (which drug, which vein)
- promote and adopt other devices in order to reduce thrombophlebitis due to the drugs (PICC?)
- keep on with monitoring vascular access.

### A45 Virtual reality as an alternative therapeutic option for the management of pain in children with Cystic Fibrosis

#### Anna Maria Iannicelli^1^, Daniele Vito^1^, Pasquale De Matteo^2^, Angela Sepe^1^, Sara Polizzi^1^, Valeria Raia^1^

##### ^1^Cystic Fibrosis Center, Department of Translational Medical Sciences, University Federico II of Naples, Naples, Italy; ^2^Division of Internal Medicine (Metabolic and Cardiac Rehabilitation Unit), Federico II University of Naples, Italy

###### **Correspondence:** Anna Maria Iannicelli (annamaria.iannicelli@unina.it)

**Background**

Virtual reality (VR) is defined by “the use of interactive simulations created with computer hardware and software to present users with opportunities to engage in environments that appear and feel similar to real world objects and events” [1]. VR quickly became a subject of study in the whole medical-therapeutic field, presenting itself as a valid alternative to Exposure Therapy [2], to improve the treatment of some pathologies and the recovery of cognitive, mental or motor functions.

**Materials and methods**

50 patients with Cystic Fibrosis aged between 8 and 18 years, were consecutively enrolled in a pilot study approved by the Ethical Committee of the University of Naples Federico II, in which VR was suggested as a tool for pain reduction during venipuncture and for anxiety reduction during intravenous antibiotics. To realize the VR, a head-mounted display and a cell phone inserted inside it were used. The display was positioned before the start of the procedure along with an oximeter, in order to investigate changes in heart rate and saturation; the display and the oximeter were removed only at the end of the procedure. The pain scale Numerical Rating Scale (NRS) was used to assess the perceived pain, while the State-Trait Anxiety Inventory questionnaire was used to assess the anxiety (S.T.A.I.). It was decided to set up the study as a case-control on the same patient. For each procedure questionnaires were administered before VR and at the end of the procedure.

**Results**

Preliminary results have shown a significant reduction in pain and anxiety when VR was used. The use of VR has also proved to be not harmful to patient safety, ensuring the completion of the procedure in safety. All but one patient who used VR reported lower pain than that reported without using it.

**Conclusions**

Although one of the most known use of VR in the scientific literature is for treating phobias and social disorders [3], VR has been demonstrated a safe method to control pain and anxiety in this cohort of patients. The reduction of the Heart Rate when the VR is used indicates a general relaxation of the patient and alienation from the procedures, which helped to perceive less pain.

**References**

1. Lee HS, Park YJ, Park SW. The Effects of Virtual Reality Training on Function in Chronic Stroke Patients: A Systematic Review and Meta-Analysis. Bio Med Res Int 2019:7595639

2. Botella C, Serrano B, Baños RM et al. Virtual reality exposure-based therapy for the treatment of post-traumatic stress disorder: a review of its efficacy, the adequacy of the treatment protocol, and its acceptability. Neuropsychiatr Dis Treat 2015;11: 2533-45

3. Oing T, Prescott J. Implementations of Virtual Reality for Anxiety-Related Disorders: Systematic Review. JMIR Serious Games 2018;6:e10965

